# Dynamic connectedness and network in the high moments of cryptocurrency, stock, and commodity markets

**DOI:** 10.1186/s40854-023-00474-6

**Published:** 2023-05-05

**Authors:** Waqas Hanif, Hee-Un Ko, Linh Pham, Sang Hoon Kang

**Affiliations:** 1 CEFAGE - Center for Advanced Studies in Management and Economics, University of Algarve, Faro, Portugal; 2 Department of Management Sciences, COMSATS University Islamabad, Attock Campus, Attock, Pakistan; 3 Korea Housing and Urban Guarantee Corporation, Busan, Republic of Korea; 4 Economics, Business and Finance Department, Lake Forest College, Lake Forest, IL 60045 USA; 5 PNU Business School, Pusan National University, Jangjeon2-Dong, Geumjeong-Gu, Busan, 609-735 Republic of Korea

**Keywords:** Spillovers, High moments, High frequency, Hedging, G14, G15

## Abstract

This study examines the connectedness in high-order moments between cryptocurrency, major stock (U.S., U.K., Eurozone, and Japan), and commodity (gold and oil) markets. Using intraday data from 2020 to 2022 and the time and frequency connectedness models of Diebold and Yilmaz (Int J Forecast 28(1):57–66, 2012) and Baruník and Křehlík (J Financ Econom 16(2):271–296, 2018), we investigate spillovers among the markets in realized volatility, the jump component of realized volatility, realized skewness, and realized kurtosis. These higher-order moments allow us to identify the unique characteristics of financial returns, such as asymmetry and fat tails, thereby capturing various market risks such as downside risk and tail risk. Our results show that the cryptocurrency, stock, and commodity markets are highly connected in terms of volatility and in the jump component of volatility, while their connectedness in skewness and kurtosis is smaller. Moreover, jump and volatility connectedness are more persistent than that of skewness and kurtosis connectedness. Our rolling-window analysis of the connectedness models shows that connectedness varies over time across all moments, and tends to increase during periods of high uncertainty. Finally, we show the potential of gold and oil as hedging and safe-haven investments for other markets given that they are the least connected to other markets across all moments and investment horizons. Our findings provide useful information for designing effective portfolio management and cryptocurrency regulations.

## Introduction

Cryptocurrencies based on blockchain technology have received considerable interest from among a wide range of stakeholders (Xu et al. 2019; Fang et al. 2022). Since the inception of the first cryptocurrency, Bitcoin, in 2009, the cryptocurrency market has experienced rapid growth and high volatility, and even more so since the start of the coronavirus (COVID-19) pandemic.^1^ As cryptocurrencies continue to be integrated into the larger financial system, it is important to understand their interlinkages with traditional investments, such as equities and commodities (Sebastião and Godinho 2021). This study examines short- and long-run spillovers among cryptocurrency, equity, and commodity investments across higher-order moments.

It is well understood that cross-market spillovers represent an important aspect of financial modeling and forecasting, asset pricing, and portfolio and risk management.^2^ Recent research has highlighted the importance of analyzing spillovers in higher-order moments via elements such as volatility, skewness, kurtosis, and the jump components of volatility, all of which can reveal useful information about asymmetry and fat-tail risks across markets (Bonato et al. 2022; Gkillas et al. 2020a; Amaya et al. 2015; Lai and Sheu 2010). This is particularly relevant when asset return distributions are generally non-normal, skewed, and prone to fat tails, a stylized fact for equity, cryptocurrency, and commodity markets (Kristjanpoller et al. 2020; Gkillas and Katsiampa 2018; Osterrierder and Lorenz 2017). The objective of this study is to analyze the interdependence among these markets in terms of realized volatility, realized skewness, realized kurtosis, and the jump component of realized volatility, and to investigate how this interdependence has evolved in response to recent global events—such as the COVID-19 pandemic, the Russia-Saudi Arabia oil price war, the cryptocurrency ban in China and other countries, and the Russo-Ukrainian War.

Numerous studies have examined spillovers among cryptocurrencies and other markets using daily data (Hasan et al. 2022; Nham et al. 2022; Pham et al. 2022; Bouri et al. 2021a; Fousekis and Tzaferi 2021; Bouri et al. 2020; Okorie and Lin 2020; Umar et al. 2021; Huynh et al. 2020; Shahzad et al. 2020; Ji et al. 2019; Symitsi and Chalvatzi 2018; Selmi et al. 2018; Ji et al. 2018; Klein et al. 2018; Bouri et al. 2017). While these analyses provide useful information about cryptocurrency behavior and its relationship with other markets, no consensus has yet been reached. Some studies have shown evidence of a weak link between cryptocurrencies and the global financial system, thereby pointing to the diversification and hedging capabilities of cryptocurrencies (Colon et al. 2021; Guesmi et al. 2019; Urquhart and Zhang 2018; Dyhrberg 2016) and safe-haven assets against downside risk (Wen et al. 2019). Others, however, show the limited abilities of cryptocurrencies as safe-haven or hedging assets (Smales 2019; Wang et al. 2019; Klein et al. 2018; Corbet et al. 2018).

Based on the above discussion, we argue that the use of daily data may have masked important information concerning the cryptocurrency market; therefore, taking advantage of high-frequency intraday data is important for characterizing the distribution of cryptocurrency at moments of greater trade volume. This study extends the literature by examining the spillover effects between cryptocurrencies and major traditional financial markets, such as equities and commodities, at higher-order moments in the return distribution. These higher-order moments reveal the unique characteristics of financial return distributions, which are typically not captured by the first-order moment (i.e., average returns). Specifically, realized volatility captures variations in returns, skewness captures the asymmetry of the return distribution, and kurtosis captures the fat tails of the return distribution. Thus, by analyzing the spillover across markets at higher-order moments, we can quantify specific market risks, such as downside risks or tail risks (He and Hamori 2021).

Specifically, we focus on the spillover effects between two major cryptocurrencies, Bitcoin and Ethereum; four major equity markets, the U.S., the U.K., the Eurozone, and Japan; and two commodities, crude oil and gold.^3^ Our sampling period ranges from January 2020 to May 2022 (inclusive), which allows us to capture the spillover effects across markets during the most recent period. Our choice of sampling period also relies on the high frequencies of extreme events during the last 2 years; for example, as with the COVID-19 pandemic in 2020, the Russia-Saudi Arabia oil price war in 2020, the cryptocurrency ban in 2021, the Russo-Ukrainian War in 2022, economic stagnation and increasing inflationary pressure in many economies due to supply chain issues, and highly volatile cryptocurrency prices. These events have had a significant impact on global financial markets, thereby highlighting the importance of studying spillovers in higher-order moments between cryptocurrencies and other markets during this period (Xiao et al. 2021; Naeem et al. 2021; Kumar et al. 2022).

Our results show a significant linkage among cryptocurrencies, commodities, and equities across moments. While volatility and jump spillovers exhibit the strongest pattern, significant spillovers still exist when we consider higher moments such as skewness and kurtosis. Moreover, we find that volatility and jump spillovers persist for a long period of time, while skewness and kurtosis spillovers dissipate quickly. These results imply that the markets are strongly linked through volatility and jump channels, while asymmetric risks (measured by realized skewness) and extreme deviations (measured by realized kurtosis) exhibit weaker spillovers. However, the analysis of skewness and kurtosis spillovers is still relevant, since our results show the switch of assets between being net transmitters and net receivers of shocks under third- and fourth-order moments, in contrast to second-order moments. This implies different behaviors of cryptocurrencies, commodities, and equities under low-probability events. Finally, we identify clusters of markets that are highly connected to one another. For example, stocks and cryptocurrencies form their own individual clusters with high connectedness within the clusters and weaker connectedness outside the clusters. This implies a hedging potential between stocks and cryptocurrencies. Moreover, Brent crude oil and gold can add diversification benefits to both stock and cryptocurrency investments because they are the least connected to other assets. Note that the diversification benefits among the assets decline significantly during crisis events—such as the COVID-19 pandemic or the Russo-Ukrainian War—as the cross-market spillovers increase significantly during such periods.

The remainder of this paper is organized as follows. “Literature review” Section presents a literature review. “Econometric modeling framework” Section discusses the methodology of this study. “Data and preliminary analysis” Section presents the data and descriptive statistics. “Empirical analysis” Section presents the empirical results. Finally, “Conclusion and policy implications” Section concludes the paper.

## Literature review

### The linkage between cryptocurrencies, equities and commodities

The relationship between cryptocurrencies and conventional assets such as equities and commodities has recently attracted substantial interest from researchers and scholars.

Previous studies have identified various relationships between cryptocurrencies and stock markets. For example, Ji et al. (2018) studied the causality between Bitcoin and the stock, bond, and commodity markets, finding that Bitcoin is isolated from other assets; however, the relationship between Bitcoin and other assets varies over time. Guesmi et al. (2019) show that a portfolio of gold, oil, emerging stock markets, and Bitcoin considerably reduces a portfolio’s risk when compared to a portfolio without Bitcoin. Wang et al. (2019) studied the linkage between 973 cryptocurrencies and 30 international equity markets and have found no evidence of the hedging capabilities of cryptocurrencies against most international indices. Bouri et al. (2020) investigated whether Bitcoin, gold, and commodities can be safe havens for various stock indices. Their findings indicate that markets are weakly dependent across different timescales and that diversification benefits vary across frequencies. Moreover, Bitcoin is superior to both gold and other commodities in hedging against risks in the stock market. Huynh et al. (2020) have analyzed the relationship between AI and robotics stocks, green bonds, and Bitcoin, and have found that a portfolio of these assets has heavy tail dependence and higher volatility transmission across the short run. They also concluded that Bitcoin and gold can hedge against one another, and that gold may be considered as a safe haven. Kristjanpoller et al. (2020) investigated the asymmetric cross-correlations between five cryptocurrencies and six equity exchange-traded funds (ETFs) and find persistence and asymmetric multifractality in these cross-correlations. Wang et al. (2022) have examined the contagion between stock markets and cryptocurrency markets, providing evidence of time-varying tail dependence, with a more significant lower tail dependence compared to upper tail dependence.

A second strand of the existing body of literature analyzes the relationship between cryptocurrency and energy investments, particularly oil prices. For example, Bouri et al. (2017) have studied the diversifying, hedging, and safe-haven properties of Bitcoin against energy commodities and found that Bitcoin is a strong hedge and safe haven against movements in energy commodities. However, hedge and safe-haven properties were only present before the 2013 Bitcoin crash. Symitsi and Chalvatzi (2018) analyzed the spillovers between Bitcoin and energy and technology investments and have thus concluded that the low correlation of Bitcoin with the energy and technology indices implies portfolio diversification benefits. Okerie and Lin (2020) have examined the volatility connectedness and hedging potential between crude oil and ten different cryptocurrencies. They have found that crude oil has short-lived hedging potential with Ethereum, and that the hedging potentials of crude oil for Solve, Elastos, and Bit Capital Vendors are long-lived. Nham et al. (2022) studied the connectedness between oil, gold, stocks, and cryptocurrencies throughout the COVID-19 pandemic, finding a significant impact by the pandemic on the connectedness among these assets.

A third strand of the literature studies the hedging and safe-haven properties of cryptocurrencies against gold. Dyhrberg’s study (2016) assesses the financial asset capabilities of Bitcoin and shows similarities to gold and the US dollar. The author concludes that Bitcoin has hedging capabilities and advantages as an exchange medium. Klein et al. (2018) compared Bitcoin and a cryptocurrency index with traditional assets and showed a completely different behavior of Bitcoin compared to gold, particularly in terms of market distress. Thus, Bitcoin is not a safe haven asset and offers no hedging capabilities in developed markets. Selmi et al. (2018) have compared the hedge, safe haven, and diversifying properties of Bitcoin and gold and have found that the abilities of gold and Bitcoin to hedge against oil prices depend on their market states and oil price movements. Smales (2019) concluded that Bitcoin is not a safe haven for other assets. Shahzad et al. (2020) have compared the safe haven, hedge, and diversification capabilities of gold and Bitcoin throughout the G7 stock markets. Their work shows that gold is a safe haven and hedge for many G7 stock indexes, whereas Bitcoin only exhibits these characteristics for Canada. Moreover, gold has higher diversification benefits and better out-of-sample hedging effectiveness than Bitcoin. Elsayed et al. (2022) studied the volatility and return connectedness of cryptocurrency, gold, and uncertainty. They found that cryptocurrency policy uncertainty is the main return spillover transmitter, whereas gold is a net receiver of both return and volatility spillovers. They concluded that gold does not provide hedging benefits against cryptocurrency uncertainty. Nakagawa and Sakemoto (2022) studied whether network factors for Bitcoin are linked to an expected return on gold, and determined a positive relationship between the expected return on gold and the number of cryptocurrency wallet users.

### High-frequency linkage between cryptocurrencies, equities and commodities

The previous subsection shows that research on the linkages between cryptocurrencies, equities, and commodities offers diverse perspectives and conclusions. For example, some studies find evidence of the potential of cryptocurrencies as hedging and safe haven investments (Colon et al. 2021; Guesmi et al. 2019; Urquhar and Zhang 2018; Dyhrberg 2016), whereas others do not (Smales 2019; Wang et al. 2019; Klein et al. 2018; Corbet et al. 2018). One possible explanation for this lack of consensus in these studies is their use of daily data, which prevents researchers from identifying the linkage between cryptocurrencies and other markets at higher moments. An analysis of market behavior at higher moments may reveal important information about the pattern of shock transmission across markets (Del Brio et al. 2017; Bouri et al. 2021b).

Recently, several studies have focused on documenting cryptocurrency behavior at higher-order moments using high-frequency intraday data. For example, Urquhart and Zhang (2019) examined the intraday hedge and safe haven properties of Bitcoin against foreign exchange rates. They find that Bitcoin is a hedge for the Swiss Franc (CHF), Euro (EUR), British Pound (GBP), a diversifier for the Australian dollar (AUD), Canadian dollar (CAD), Japanese Yen (JPY), and a safe haven for CAD, CHF and GBP. Ahmed (2020) investigated the risk-return tradeoff in Bitcoin and found no evidence to support the risk-return tradeoff hypothesis in the Bitcoin market. Yousaf and Ali (2020) explored the linkage among major cryptocurrencies using high-frequency data and found varying correlations between cryptocurrencies before and after the COVID-19 pandemic. Yarovaya et al. (2021) investigated herding behavior in cryptocurrency markets in response to the COVID-19 pandemic. Ahmed (2021) has studied the impact of the second moment in cryptocurrency markets (i.e., volatility) on Islamic equity markets. While these studies utilize high-frequency data, they focus on spillovers across cryptocurrencies at the first- and second-order moments (returns and volatility).

With respect to the behavior of cryptocurrencies at higher moments (e.g., skewness and kurtosis), recent studies have analyzed the impact of these higher moments within cryptocurrency markets. Hasan et al. (2021) examined higher-moment connectedness among three dominant cryptocurrencies and identified moderate realized volatility connectedness, robust realized skewness connectedness, and strong realized kurtosis connectedness among the cryptocurrencies. Nagy and Benedek (2021) have illustrated that the Sharpe ratios of cryptocurrencies are influenced by higher co-moments of returns. Ahmed and Al Mafrachi (2021) examined the sensitivity of cryptocurrency returns to higher order realized moments. Jia et al. (2021) analyzed the cross-sectional return predictability of the higher moments of 84 cryptocurrencies and found strong evidence of a positive relationship between volatility and kurtosis with returns and a negative relationship between skewness and returns. Bouri et al. (2021c) discovered the role of the US-China trade war on the forecast ability of cryptocurrency, controlling for higher moments of return distributions. Ma and Luan (2022) analyzed the effect of Ethereum synchronicity and found that it is a new factor in Bitcoin pricing and is highly correlated with Bitcoin crash risk in an uptrend. In contrast, most equity and commodity returns have no significant link to Bitcoin crash risk. Kakinaka and Umeno (2022) have shown asymmetric spillovers across the cryptocurrency markets.

However, little attention has been paid to the spillovers between cryptocurrencies and other financial markets at the third and fourth moments (skewness and kurtosis). Gkillas et al. (2020b) analyzed the spillover in jumps and realized identified second, third, and fourth moments among crude oil, gold, and Bitcoin markets using Granger causality and generalized impulse response analysis. They found evidence of linkages across markets at all moments and emphasize the importance of modeling cryptocurrency markets at higher moments. Hou et al. (2022) have analyzed the higher-moment spillovers between Bitcoin and the crude oil market and have found evidence of a regime switch in the Bitcoin-oil relationship after the US-China trade war.

The objective of this study is to examine the spillover effects between cryptocurrency (as an asset class and store of wealth) and major traditional financial markets, such as equities and commodities at higher-order moments, specifically addressing realized volatility, skewness, kurtosis, and the jump component of realized volatility. Our sample spans the period from January 2020 to May 2022 and includes two major cryptocurrencies, Bitcoin and Ethereum; four major equity markets, the U.S., the U.K., the Eurozone, and Japan; and two commodities, crude oil and gold. Using the time and frequency connectedness framework of Diebold and Yilmaz (2012) and Baruník and Křehlík (2018),^4^ our empirical results show that connectedness across markets changes significantly between moments and between the short and long run.

Our study contributes to the literature in several ways. First, we analyze spillovers across a wide range of moments (volatility, skewness, kurtosis, and jump elements of volatility) across a wide range of markets, including major cryptocurrency, stock, and commodity markets. Second, using a frequency connectedness network analysis, we identify multivariate interlinkages among these markets across moments and frequencies. This allows us to capture the direct and indirect transmission of shocks across markets and to study how these transmission patterns evolve between the short and long run. Finally, by focusing on the period after 2020, we capture the behavior of cryptocurrency markets during a period with many extreme events. In short, by employing high-frequency data for a wide range of assets in a multivariate setting, our study offers a more detailed view of the linkages between cryptocurrencies and other markets at different times and frequencies. These provide market participants with true intraday market dynamics between cryptocurrencies and other markets, and have important implications for effective risk management.

## Econometric modeling framework

Market connectedness in higher-order moments (second, third, and fourth moments) offers new insights to market participants about the evolution of interconnections among different assets. Controlling extreme deviations from the mean returns (fat tails and jumps) improves the quantity and quality of information flows. In this study, we first construct high-order moments (realized volatility, jump volatility, realized skewness, and realized kurtosis) using 5-min data. Second, we estimate the total, directional, and net connectedness using the DY12 and BK18 approaches.^5^ Finally, the connectedness network is illustrated at different frequencies (total, short-term, and long-term horizons) to understand the transmission path of information across different assets.^6^

### Realized variances, skewness and kurtosis

We measure volatility spillover—as suggested by Barndorff-Nielsen et al. (2010) and Barunik et al. (2015, 2017)—to identify asymmetries due to negative and positive shocks. Based on the literature (Andersen and Bollerslev 1998), “realized variance” $\left( {RV_{t} } \right)$ is the square of the return series estimated at every 5-min interval. The realized variance can be expressed as

1 $$RV_{t} = \mathop \sum \limits_{s = 1}^{N} r_{s,t}^{2} , t = 1,2, \ldots .,T$$

where ${\text{s}} = 1, \ldots ,{\text{N}}$ is an observation; that is, a 5-min interval frequency of the return series $\left( {r_{s,t} } \right)$.

Next, we detect jumps in realized volatility. While realized volatility captures the average dispersion of financial returns, the threshold bi-power variation (TBPV) jump component of volatilities captures the discontinuities in volatility, which have been shown to play a significant role in improving realized moment forecasts (e.g. Lai and Sheu 2010; Gkillas et al. 2019, 2020a). Using the TBPV of Corsi et al. (2010), we calculate the jump statistic $J_{t}^{{\left( {TBPV} \right)}}$ as follows:

2 $$J_{t}^{{\left( {TBPV} \right)}} = \sqrt T \frac{{\left( {RV_{t} - TBPV_{t} } \right)RV_{t}^{ - 1} }}{{\left[ {\left( {\zeta_{1}^{ - 4} + 2\zeta_{1}^{ - 2} - 5} \right)max\left\{ {1,TQ_{t} TBPV_{t}^{ - 2} } \right\}} \right]^{1/2} }}$$

where $\zeta_{1}^{{}} = \sqrt {2/\pi }$; $TQ_{t} = T\zeta_{4/3}^{ - 3} \sum\nolimits_{s = 1}^{N} {\left| {r_{t,s} } \right|^{4/3} \left| {r_{t,s + 1} } \right|^{4/3} \left| {r_{t,s + 2} } \right|^{4/3} }$ is the realized tri-power quarticity and convergence in the probability of integrated quarticity. The threshold bi-power variation $\left( {TBPV_{t} } \right)$ as a jump-free volatility estimator is expressed as follows:

3 $$TBPV_{t} = \mathop \sum \limits_{s = 2}^{N} \left| {r_{t,s - 1} } \right|, \left| {r_{t,s} } \right|I_{{\left\{ {\left| {r_{t,s - 1} } \right|^{2} \le \theta_{i - 1} } \right\}}} I_{{\left\{ {\left| {r_{t,s - 1} } \right|^{2} \le \theta_{i} } \right\}}} ,s$$

where $I_{{\left\{ \cdot \right\}}}$ represents an indicator function; $r_{t,s}$ is the intraday return series; and ${\Theta }$ is the threshold function. The jump statistic $J_{t}^{{\left( {TBPV} \right)}}$ follows the appropriate critical value of the standard Gaussian distribution. Further, we define the jump component of realized volatility in daily frequency as follows:

4 $$J_{t} = \left| {RV_{t} - TBPV_{t} } \right|I_{{\left\{ {J_{t}^{{\left( {TBPV} \right)}} > {\Omega }_{\alpha } } \right\}}} ,$$

where $I_{{\left\{ \cdot \right\}}}$ is an indicator function of $J_{t}^{{\left( {TBPV} \right)}}$ exceeding a given critical value of a Gaussian distribution denoted ${\Omega }_{\alpha }$, at the ${\upalpha }$ significant level.

While the second moment (realized volatility) includes both jumps and continuous components, the third and fourth moments (realized skewness and kurtosis) only include the jump parameters. Specifically, the second moment converges to quadratic variation, whereas the third and fourth moments do not converge to their respective cubic and quartic variations (Gkillas et al. 2020a). Following Bouri et al. (2021b) and Gkillas et al. (2020a), we further calculate two high moments (skewness and kurtosis) to analyze the jump risk spillover. First, skewness measures the asymmetry of the conditional asset return distribution as a proxy for asymmetric risk (Barndorff-Nielsen et al. 2010). A negative (positive) value indicates a left-skewed distribution (right-skewed distribution), in which the left (right) tail of the distribution is longer or fatter than the tail on the right (left) side. The realized skewness $\left( {RS_{t} } \right)$ captures the continuous component of the cubic variation and is related to the jump contribution (Amaya et al. 2015). The daily realized skewness $\left( {RS_{t} } \right)$ can be expressed as

5 $$RS_{t}^{{}} = \frac{{\sqrt N \mathop \sum \nolimits_{i = 1}^{N} r_{t,s}^{3} }}{{RV_{t}^{3/2} }}$$

Second, kurtosis—a measure of “tailedness” of the conditional asset return distribution—corresponds to the extremity of deviations (Barndorff-Nielsen and Shephard 2004). The daily realized kurtosis $\left( {RK_{t} } \right)$ is is constructed as follows:

6 $$RK_{t}^{{}} = \frac{{N\mathop \sum \nolimits_{i = 1}^{N} r_{t,s}^{4} }}{{RV_{t}^{2} }}$$

where $RK_{t}$ captures the discontinue component of quadratic variation.

### The Diebold and Yilmaz method

We first discuss the DY12 methodology (Diebold and Yilmaz 2012), which is based on a vector autoregressive (VAR) model. The focus is to compute the generalized forecast error variance decompositions (FEVD) from a VAR model. Consider a variance-stationary *n*-variable VAR(p)

7 $$y_{t} = \mathop \sum \limits_{i = 1}^{p} {\Phi }_{i} y_{t - 1} + \varepsilon_{t} ,$$

where ${ }\varepsilon_{t} \sim N\left( {0,{\Sigma }} \right)$, $y_{t}$ is an $n*1$ vector of endogenous variables, and ${\Phi }_{i} ,$ represents the $n*n$ autoregressive coefficient matrices. The moving average (MA) representation of this model can be rewritten as

8 $$y_{t} = \mathop \sum \limits_{j = 0}^{\infty } {\Psi }_{j} \varepsilon_{t} ,$$

where ${\Psi }_{j}$ is an $n*n$ coefficient matrix in line with the recursion of the form, ${\Psi }_{j} = {\Phi }_{1} {\Psi }_{j - 1} + {\Phi }_{2} {\Psi }_{j - 2} + \ldots + {\Phi }_{p} {\Psi }_{j - p}$, where ${\Psi }_{0}$ is the $n*n$ identity matrix and ${\text{A}}_{j} = 0 {\text{for}} j < 0.$ Tackling the problem of orthogonal innovation, DY12 utilizes the generalized VAR set up for the Koop et al. (1996) (hereafter, KPPS) H-step forecast variance for H = 1, 2…n, given as

9 $$\left( {\Theta_{H} } \right)_{j,k} = \frac{{\sigma_{kk}^{ - 1} \mathop \sum \nolimits_{h = 0}^{H} \left( {\left( {\Psi_{h} \Sigma } \right)_{j,k} } \right)^{2} }}{{\mathop \sum \nolimits_{h = 0}^{H} \left( {\Psi_{h} \Sigma \Psi_{h}^{^{\prime}} } \right)_{j,j} }},$$

where $\Psi_{h}$ is an $n \times n$ matrix of coefficients corresponding to lag $h$, and $\sigma_{kk} = \left( \Sigma \right)_{k,k}$. The term $\left( {\Theta_{H} } \right)_{j,k}$ denotes the contribution of the $k$th variable of the system to the variance of the forecast error of element *j*. In the generalized VAR framework, the shocks to each variable are not orthogonalized; thus, the sum of each row of $\left( {\Theta_{H} } \right)_{j,k}$ are not generally equal. Therefore, each decomposition matrix element can be normalized by dividing by the row sum, as follows:

10 $$\left( {\Theta_{H} } \right)_{j,k} = \frac{{\left( {\Theta_{H} } \right)_{j,k} }}{{\mathop \sum \nolimits_{k = 1}^{n} \left( {\Theta_{H} } \right)_{j,k} }},\,\,\,{\text{with}}\,\,\,\mathop \sum \limits_{k = 1}^{n} \left( {\tilde{\Theta }_{H} } \right)_{j,k} = 1 \,\,{\text{and}}\,\,{ }\mathop \sum \limits_{i,k = 1}^{n} \left( {\tilde{\Theta }_{H} } \right)_{j,k} = N$$

Thus, the connectedness measure is the share of variances in the forecasts generated by factors other than forecast errors or, equally, the sum of the off-diagonal elements to the sum of the whole matrix (Diebold and Yilmaz 2012)

11 $$C_{H} = 100 \times \frac{{\mathop \sum \nolimits_{j \ne k} \left( {\tilde{\Theta }_{H} } \right)_{j,k} }}{{\sum \left( {\tilde{\Theta }_{H} } \right)_{j,k} }} = 100 \times \left( {1 - \frac{{Tr\left\{ {\tilde{\Theta }_{H} } \right\}}}{{\sum \left( {\tilde{\Theta }_{H} } \right)_{j,k} }}} \right),$$

where $Tr \left\{ \cdot \right\}$ denotes the trace operator. Hence, connectedness is the relative contribution of the other variables in the system to the forecast variances. $C_{H}$ measures the connectedness of the entire system. Furthermore, we can also measure the directional spillovers received by market $j$ from all other markets, $k$, and vice versa. The net volatility spillovers from each market to all other markets is the difference between the directional spillovers received from the markets and those that contributed to the market.

### Frequency spillover method

We now elaborate on the frequency-domain spillover index of Baruník and Křehlík (2018; hereafter, the BK18 approach) for measuring connectedness. As seen in Eq. (3), the connectedness measure is based on the impulse function ${\Psi }_{h}$ defined in the time domain. Let us consider a frequency response function ${\Psi }\left( {e^{ - iw} } \right) = \mathop \sum \limits_{h} e^{ - iwh} {\Psi }_{h}$, which can be obtained from the Fourier transform of coefficient ${\Psi }$ with ${\text{i}} = \sqrt { - 1}$. The generalized causation spectrum over frequencies $\omega = \in \left( { - \pi ,\pi } \right)$ is specified as

12 $$\left( {f\left( \omega \right)} \right)_{j,k} \equiv \frac{{\sigma_{kk}^{ - 1} \left| {\left( {\Psi \left( {e^{ - iw} } \right)\Sigma } \right)_{j,k} } \right|^{2} }}{{\left( {\Psi \left( {\omega^{ - iw} } \right)\Sigma \Psi^{\prime } \left( {e^{ + iw} } \right)} \right)_{j,j} }},$$

where ${\Psi }\left( {e^{ - iw} } \right)$ is the Fourier transform of impulse response ${\Psi }$. Note that $\left( {f\left( \omega \right)} \right)_{j,k}$ represents the portion of the spectrum of the $j$th variable at frequency ω owing to shocks to the $k$th variable. Thus, we can interpret the quantity as a within-frequency causation because the denominator holds the $j$th variable spectrum—the diagonal elements of the cross-spectral density of $x_{i}$, at a given frequency ${\upomega }$. We can weigh $\left( {f\left( \omega \right)} \right)_{j,k}$ by the frequency share of the variance of the $j$th variable to obtain a natural decomposition of the original generalized FEVD into frequencies. We define the weighting function as

13 $$\Gamma_{j} \left( \omega \right) = \frac{{\left( {\Psi \left( {e^{ - iw} } \right)\Sigma \Psi^{\prime } \left( {e^{ + iw} } \right)} \right)_{j,j} }}{{\frac{1}{2\pi }\mathop \int \nolimits_{ - \pi }^{\pi } \left( {\Psi \left( {e^{ - i\lambda } } \right)\Sigma \Psi^{\prime } \left( {e^{ + i\lambda } } \right)} \right)_{j,j} d\lambda }},$$

where the power of the $j$th variable at a given frequency sums the frequencies to a constant value of $2\pi$. Although the Fourier transformation of the impulse response is generally a complex-valued quantity, the generalized causation spectrum is the squared modulus of the weighted complex numbers and, hence, produces a quantity. Frequency band $d$ is defined as: $d = \left( {a,b} \right):a,b \in \left( { - \pi ,\pi } \right),a.$ Consequently, the generalized FEVD on frequency band *d* can be expressed as

14 $$\left( {{\Theta }_{d} } \right)_{j,k} = \frac{1}{2\pi }\mathop \int \limits_{d}^{{}} {\Gamma }_{j} \left( \omega \right)\left( {f\left( \omega \right)} \right)_{j,k} d\omega .$$

Using the generalized FEVD spectral representation, the connectedness can be described in a given frequency band. We define the scaled generalized FEVD on the frequency band $d$ as as

15 $$\left( {{\tilde{\Theta }}_{d} } \right)_{j,k} = \frac{{\left( {{\Theta }_{d} } \right)_{j,k} }}{{\mathop \sum \nolimits_{k} \left( {{\Theta }_{\infty } } \right)_{j,k} }}$$

The frequency connectedness on the frequency band $d$ is then defined as:

16 $$C_{d}^{F} = 100 \times \left( {\frac{{\mathop \sum \nolimits_{j \ne k} \left( {{\tilde{\Theta }}_{d} } \right)_{j,k} }}{{\sum \left( {{\tilde{\Theta }}_{\infty } } \right)_{j,k} }} - \frac{{Tr\left\{ {{\tilde{\Theta }}_{d} } \right\}}}{{\sum \left( {{\tilde{\Theta }}_{\infty } } \right)_{j,k} }}} \right)$$

Finally, the overall connectedness within the frequency band $d$ can be defined as:

17 $$C_{d}^{W} = 100 \times \left( {1 - \frac{{Tr\left\{ {{\tilde{\Theta }}_{d} } \right\}}}{{\sum \left( {{\tilde{\Theta }}_{d} } \right)_{j,k} }}} \right)$$

It is also worth noting that the within-connectedness value provides the connectedness effect that occurs within the frequency band, and is weighted exclusively by the power of the series on the given frequency band. Conversely, frequency connectedness decomposes the original connectedness into separate parts that provide the original connectedness measure when summed.

In summary, our empirical analysis consists of three steps. First, by using intra-day data, we compute the realized higher-order moments (variance, skewness, and kurtosis). Next, we compute the time-domain connectedness indexes using the DY12 approach. Then, by following BK 18, we use spectral decomposition to decompose the time-domain connectedness indexes into different frequency bands to study how the connectedness among the markets changes over different horizons. In this study, we define the short term as the spectral decomposition of the original GFEVD over the 1–5 days frequency band (a trading week) and the long term as the spectral decomposition of the original GFEVD over the 6 + day frequency band.

## Data and preliminary analysis

This study considers two cryptocurrencies, Bitcoin (BTC) and Ethereum (ETH); two commodities, Brent crude oil (BRENT) and gold (GOLD); and four stock markets, the Eurozone, the U.K., Japan, and the U.S. We use the EUROSTOXX50, FTSE100, NIKKEI225, and SandP500 indexes as proxies for the Eurozone, London, Japan, and U.S. stock markets. In subsequent analyses, these indexes are abbreviated as EUROSTOXX50, FTSE100, NIKKEI225, and SP500, respectively. The data were sampled at 5-min interval from 0:00 a.m. to 23:55 p.m. (GMT) for the period from January 1, 2020, to May 31, 2022. High-frequency data used in this study were obtained from Bloomberg.^7^

**Fig. 1:**
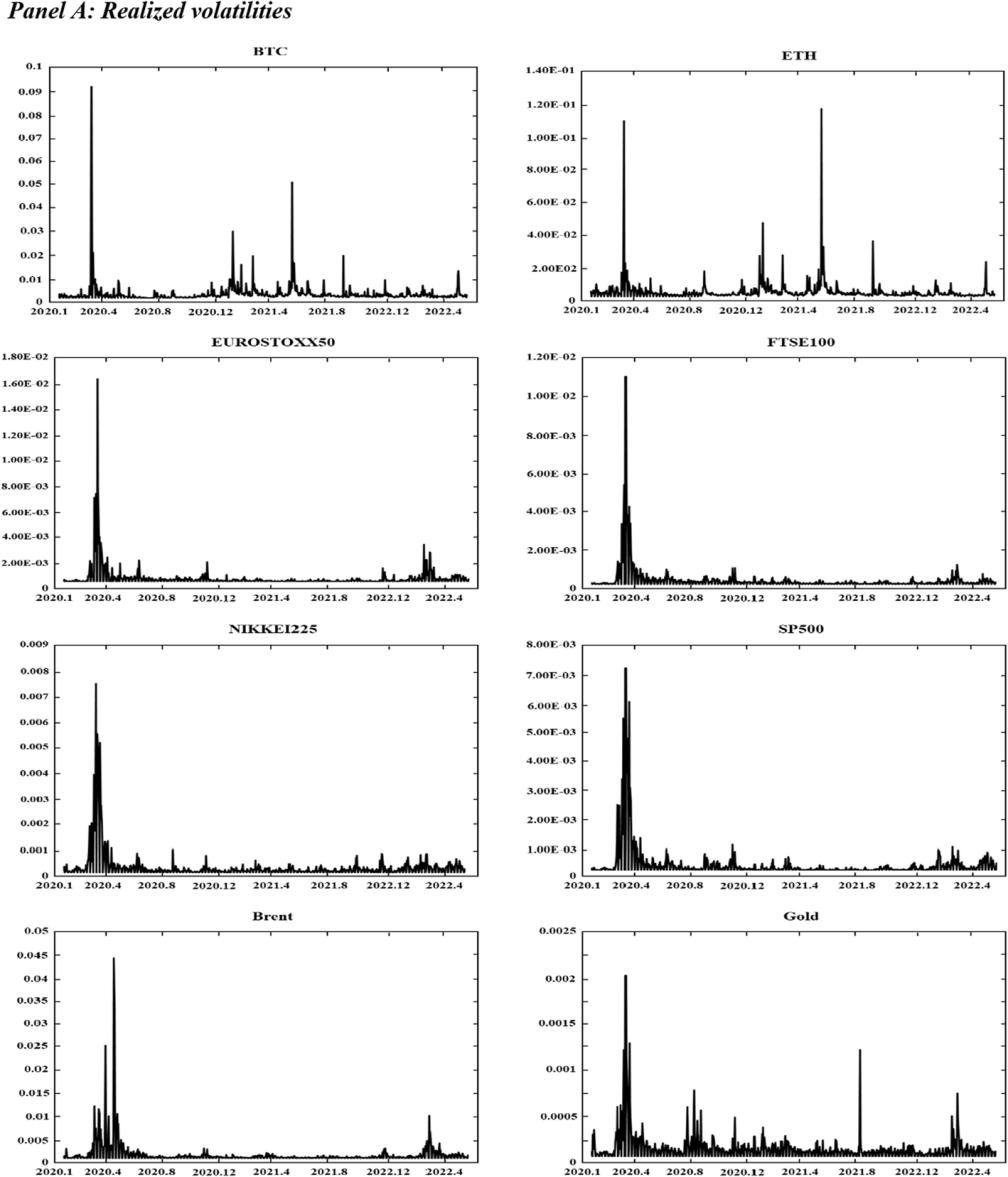

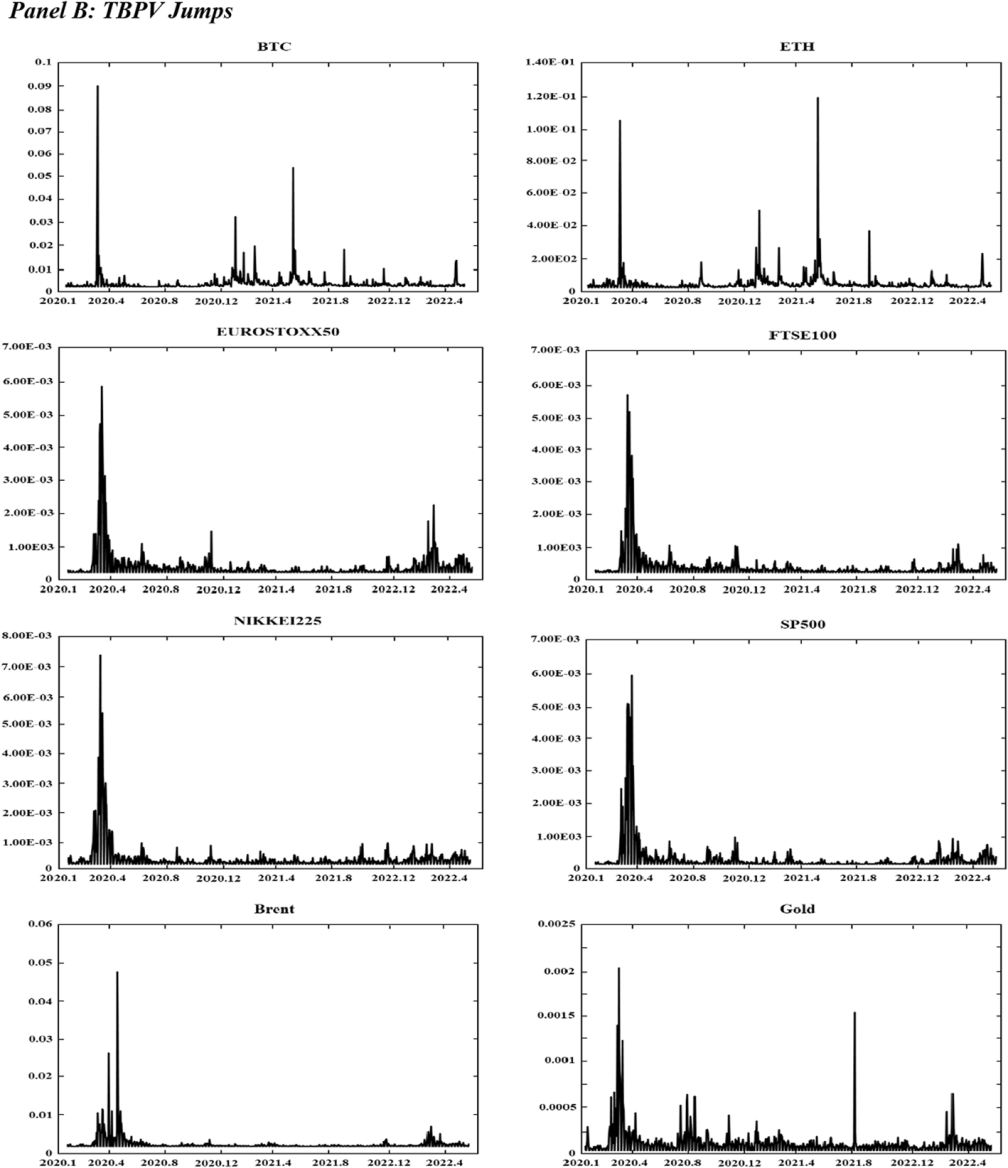

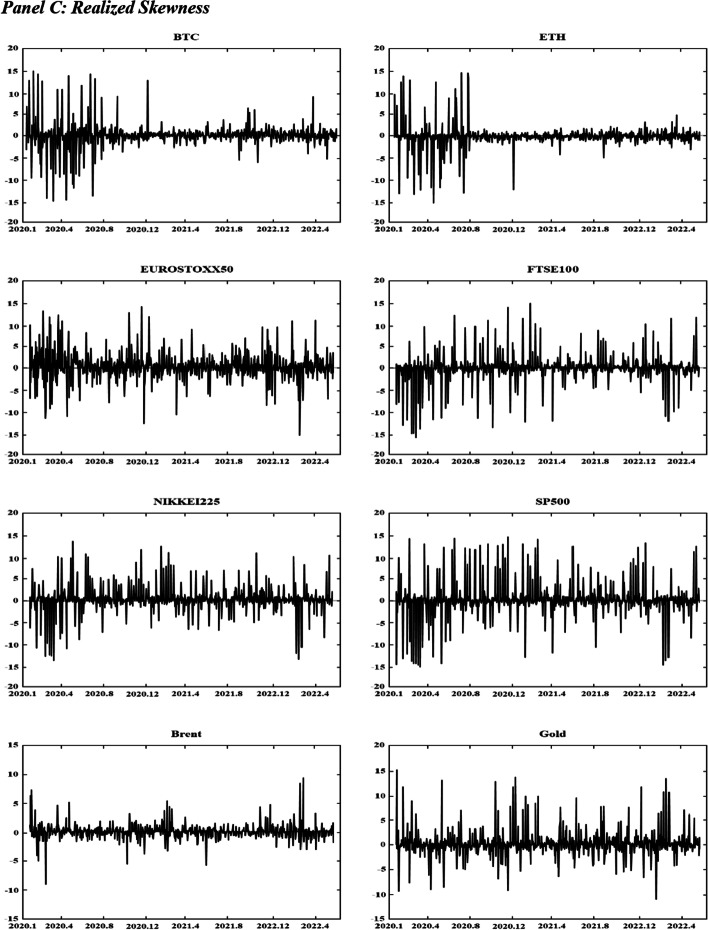

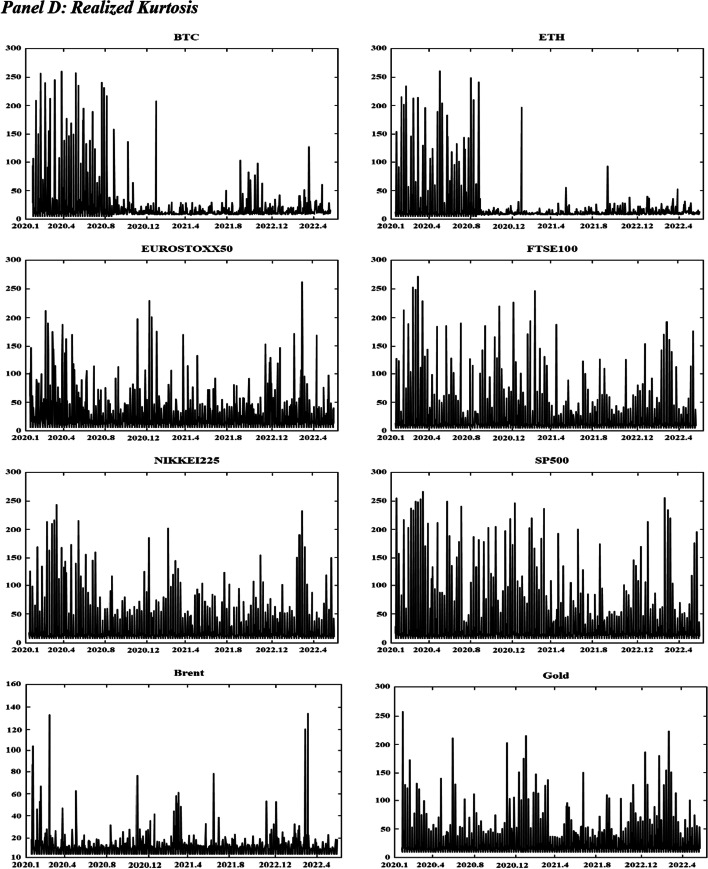
Dynamics of realized volatilities, jumps, skewness and kurtosis

Figure 1 displays the time variations in the realized volatilities, TBPV jumps, realized skewness, and realized kurtosis of the assets. T shows an increase in the realized volatility and jump measures for the two cryptocurrencies (BTC and ETH) at the beginning of 2020 and in mid-2021. These periods are characterized by the COVID-19-induced financial crisis (early 2020) and the tightening of cryptocurrency regulations in many countries (mid 2021).^8^ We have observed an increase in realized volatilities and jumps in all other markets at the start of the COVID-19 pandemic (early 2020) and at the beginning of 2022, which coincides with the onset of the Russo-Ukrainian War. Regarding the skewness measures, Fig. 1 shows substantial fluctuations in the skewness measures among all assets, and the realized kurtosis measures show evidence of fat tails among all markets throughout our sampling periods. These observations are consistent with the increasing uncertainty during our sampling periods, which can be attributed to many different events (e.g., the different phases of COVID-19 and their associated regulations, the Russia-Saudi Arabia oil price war, and the Russo-Ukrainian War).

Table 1 summarizes the descriptive statistics for cryptocurrency, commodities, and stocks’ realized volatility, kurtosis, skewness, and jumps. On average, Brent crude oil experiences the highest realized volatility (0.00075), whereas gold experiences the lowest realized volatility (8.5273e−005). The average realized volatility of the other markets ranges from 0.00018 to 0.00031. Similarly, crude oil experienced more jumps than other markets during the sampling period. This is consistent with the fact that the period under study (2020–2022) is characterized by large volatilities in oil prices. Our realized skewness measures show that all markets are positively skewed, on average, except for Ethereum. The skewness measure is the largest for gold, which reflects investors’ preference for gold as a safe haven asset during the period of 2020–2022. The kurtosis measures show that all assets have thicker tails than that of normal distribution. The Jarque–Bera tests show that the realized volatility, kurtosis, skewness, and jumps are not normally distributed, whereas the Ljung Box test statistics show evidence of volatility clustering across all variables. Finally, the ERS test statistics show that all the series are stationary. The results of the ADF unit root test reject the null hypothesis of the unit root. Therefore, these series can be used in the spillover models described in “Econometric modeling framework” Section.

**Table 1:** Descriptive statistics for cryptocurrency, commodity and stock’s realized volatility, kurtosis, skewness, and jumps

	BTC	ETH	EURO STOXX50	FTSE100	NIKKEI225	SP500	Brent	Gold
*Panel A: realized volatility*								
Mean	0.0018	0.0031	0.00024	0.00019	0.00021	0.00018	0.00075	8.5273e−005
Std.dev	0.0046	0.0068	0.00082	0.00059	0.00056	0.00056	0.00243	0.00014
Skewness	13.016***	11.500***	11.945***	11.092***	7.537***	7.785***	11.605***	6.370***
Kurtosis	219.28***	169.88***	194.84***	165.94***	69.19***	72.10***	174.62***	59.57***
Jarque–Bera	1,790,023.***	1,078,843.***	1,414,581.***	1,028,888.***	184,104.***	199,726.***	1,139,196.***	136,224.***
ERS	− 8.598***	− 8.866***	− 7.406***	− 5.867***	− 4.710***	− 4.912***	− 8.966***	− 7.060***
ADF	− 10.195***	− 9.934***	− 8.283***	− 6.803***	− 5.303***	− 5.015***	− 11.129***	− 7.791***
Q(10)	258.77***	280.29***	799.24***	1072.58***	1822.03***	1671.85***	492.23***	692.52***
Q2(10)	78.35***	25.65***	133.69***	214.04***	960.52***	846.35***	172.90***	316.74***
*Panel B: TBPV jumps*								
Mean	0.0016	0.0027	0.00018	0.00017	0.00018	0.00016	0.00069	8.1013e−005
Std.dev	0.0046	0.0068	0.00045	0.00044	0.00050	0.00051	0.0024	0.00014
Skewness	13.18***	11.80***	7.499***	7.884***	8.191***	7.629***	12.89***	6.964***
Kurtosis	219.54***	176.23***	70.06***	74.55***	84.18***	66.73***	213.07***	67.82***
Jarque–Bera	1,794,802.***	1,160,643.***	188,445.***	213,150.***	270,034.***	172,015.***	1,690,984.***	176,001.***
ERS	− 9.093***	− 9.140***	− 6.135***	− 5.864***	− 5.783***	− 5.778***	− 9.167***	− 7.304***
ADF	− 12.315***	− 9.931***	− 4.541***	− 4.494***	− 4.778***	− 4.703***	− 3.362**	− 4.705***
Q(10)	233.56***	257.70***	1171.68***	1300.32***	1407.31***	1363.90***	449.89***	583.27***
Q2(10)	72.48***	23.07***	824.05***	794.84***	693.21***	850.15***	125.40***	228.37***
*Panel C: realized skewness*								
*Mean*	0.21	− 0.073	0.005	0.069	0.024	0.001	0.013	0.122
Std.dev	8.94	8.732	7.705	13.459	7.122	5.518	1.448	6.359
Skewness	0.216***	− 0.808***	− 0.343***	0.043	0.255***	0.041	0.910***	1.615***
Kurtosis	5.888***	11.13***	9.410***	8.162***	15.73***	23.34***	18.41***	10.85***
Jarque–Bera	1230.***	4469.***	3141.***	2351.***	8800.***	19,234.***	12,084.***	4523.***
ERS	− 2.947***	− 12.87***	− 12.47***	− 13.37***	− 4.134***	− 12.49***	− 5.342***	− 13.54***
ADF	− 3.501***	− 2.849**	− 3.794***	− 4.974***	− 4.578***	− 5.087***	− 9.544***	− 4.925***
Q(10)	4.245	2.228	8.147	4.633	16.43***	5.597	13.83***	10.02*
Q2(10)	30.30***	50.74***	75.26***	84.10***	106.47***	92.99***	21.65***	48.46***
*Panel D: Realized kurtosis*								
Mean	16.71	12.96	23.67	18.48	18.05	23.64	7.346	17.84
Std,dev	1239.29	1039.07	1162.72	1513.32	1211.91	2383.6	144.11	998.56
Skewness	4.762***	5.450***	3.120***	3.740***	3.476***	3.241***	6.065***	3.697***
Kurtosis	24.29***	30.956***	11.986***	15.014***	13.306***	10.207***	49.843***	16.287***
Jarque–Bera	24,993.***	39,539.***	6702.***	10,327.***	8273.***	5367.***	96,594.***	11,744.***
ERS	− 11.37***	− 12.87***	− 5.302***	− 14.35***	− 15.96***	− 14.42***	− 8.336***	− 11.36***
ADF	31.427***	− 5.438***	− 29.326***	− 29.427***	− 28.786***	− 29.658***	− 30.038***	− 29.938***
Q(10)	116.82***	119.86***	55.86***	202.49***	266.95***	254.10***	44.30***	227.57***
Q2(10)	74.17***	75.45***	26.07***	85.34***	152.90***	153.86***	25.42***	82.58***

*** and ** indicate significance at the 1% and 5% level, respectively. Jarque–Bera stands for the Jarque–Bera test for the null hypothesis of a normal distribution. ERS stands for the Elliott–Rothenberg–Stock test of stationarity, where the null hypothesis is that the series follows a random walk. ADF indicates the unit root test of Dickey–Fuller (1979) which checks the null hypothesis of unit root for the residuals. Q(10) and Q2(10) stand for the Ljung-Box tests on the original series and its squared terms

## Empirical analysis

### Static spillovers analysis

Table 2 reports the static connectedness in realized volatility, jumps, skewness and kurtosis among cryptocurrencies, commodities, and stocks. Note that the static connectedness table is estimated via the DY12 model with a 200-day window and lag length of order 1 (BIC) and a 10-step-ahead forecast. Each cell in the table represents the percent of forecast error variance in the row variable that is explained by the column variable. The column “FROM” indicates the total connectedness received by each market from the whole system. The row “TO” indicates the total connectedness transmitted by each market to the whole system. The row “NET” shows the net connectedness of each market, where a positive (negative) value indicates whether a market is a net shock transmitter (receiver). “TCI” indicates the value of total connectedness index, which captures the overall degree of connectedness in the system.

**Table 2:** Realized estimates connectedness table

	BTC	ETH	EURO STOXX 50	FTSE100	NIKKEI 225	SP500	Brent	Gold	FROM
*Panel A: realized volatility*									
BTC	36.47	30.84	5.99	6.31	7.27	6.04	3.15	3.93	63.53
ETH	28.96	37.66	5.15	6.12	8.37	6.74	3.26	3.74	62.34
EURO STOXX50	2.46	2.56	25.54	22.79	13.36	16.28	10.49	6.52	74.46
FTSE100	2.54	2.85	19.58	25.46	14.07	18.55	10.22	6.73	74.54
NIKKEI225	2.78	3.1	15.81	18.16	28.08	15.01	9.67	7.38	71.92
SP500	2.58	3.51	17.39	21.98	14.6	25.9	8.13	5.91	74.1
Brent	2.96	3.51	15.33	15.61	8.87	9.84	36.88	6.99	63.12
Gold	4.41	4.25	10	11.6	8.2	8.04	7.1	46.41	53.59
TO	46.69	50.63	89.25	102.5	74.74	80.51	52.03	41.19	537.6
Own	83.16	88.29	114.7	128.0	102.82	106.41	88.91	87.6	TCI
NET	− 16.84	− 11.71	14.78	28.03	2.82	6.41	− 11.09	− 12.4	67.20
*Panel B: TBPV jumps*									
BTC	37.43	31.58	5.52	5.4	6.72	5.65	3.5	4.2	62.57
ETH	30.08	38.98	5.15	4.96	7.06	6.31	3.08	4.37	61.02
EURO STOXX50	2.73	2.91	23.92	21.09	13.96	17.59	9.92	7.9	76.08
FTSE100	2.94	3.24	21.17	22.68	14.19	17.86	10.26	7.67	77.32
NIKKEI225	3.32	3.37	17.07	16.76	25.49	15.69	10.27	8.04	74.51
SP500	2.77	3.72	19.1	19.51	14.39	24.85	8.62	7.04	75.15
Brent	2.75	2.89	14.18	14.43	10.93	10.31	36.35	8.16	63.65
Gold	5.12	4.51	10.52	10.01	7.75	7.98	6.74	47.36	52.64
TO	49.72	52.22	92.71	92.15	74.99	81.39	52.39	47.37	542.95
Own	87.15	91.2	116.63	114.83	100.48	106.24	88.74	94.73	TCI
NET	− 12.85	− 8.8	16.63	14.83	0.48	6.24	− 11.26	− 5.27	67.87
*Panel C: realized skewness*									
BTC	70.26	24.31	0.9	1.15	0.59	1.11	0.89	0.78	29.74
ETH	23.23	70.96	1.13	1.31	0.9	1.27	0.28	0.91	29.04
EURO STOXX50	0.78	0.93	60.39	14.51	9.22	11.73	0.31	2.12	39.61
FTSE100	0.73	0.75	7.98	51.26	13.5	24.43	0.14	1.22	48.74
NIKKEI225	0.42	0.72	3.76	13.73	55.11	23.16	0.15	2.95	44.89
SP500	0.79	1.11	5.55	22.37	20.61	47.27	0.1	2.21	52.73
Brent	1.65	1.61	2.5	5.39	4.23	3.7	78.59	2.35	21.41
Gold	0.98	0.91	1.56	1.93	4.3	3.88	0.24	86.2	13.8
TO	28.58	30.34	23.37	60.39	53.36	69.29	2.12	12.53	279.98
Own	98.83	101.29	83.76	111.65	108.47	116.55	80.71	98.74	TCI
NET	− 1.17	1.29	− 16.24	11.65	8.47	16.55	− 19.29	− 1.26	35.00
*Panel D: realized kurtosis*									
BTC	57.22	26.31	2.92	2.77	3.44	1.92	2.45	2.95	42.78
ETH	25.49	54.47	2.94	3.9	4.49	2.46	2.52	3.72	45.53
EURO STOXX50	4.41	4.26	56.62	7.9	6.83	6.47	5.09	8.42	43.38
FTSE100	2.49	3.08	5.71	48.36	12.03	17.54	3.53	7.26	51.64
NIKKEI225	3.15	2.82	3.62	9.08	48.44	18.89	4.77	9.24	51.56
SP500	2.14	2.16	3.77	11.83	13.75	46.55	6.5	13.3	53.45
Brent	3.1	3.13	5.24	4.47	5.51	4.33	70.43	3.79	29.57
Gold	3.95	4.14	4.94	7.17	10.56	11.08	6.24	51.92	48.08
TO	44.73	45.9	29.15	47.13	56.61	62.69	31.1	48.68	365.98
Own	101.95	100.37	85.78	95.49	105.04	109.24	101.53	100.6	TCI
NET	1.95	0.37	− 14.22	− 4.51	5.04	9.24	1.53	0.6	45.75

The column “FROM” indicates the total connectedness received by the market i from other variables in the whole system. The row “TO” indicates the total connectedness transmitted by the market i to the whole system, excluding itself. The row “Own” indicates the total connectedness transmitted by the market i to the system, including itself. The row “NET” shows the net connectedness of each market. “TCI” indicates the value of the total connectedness index. Each cell corresponds to the spillover from the column variable to the row variable

Panel A shows that the average realized volatility spillover index is 67.20%, with varying degrees of connectedness across markets. Specifically, the two cryptocurrencies (BTC and ETH) are highly connected to each other with cross-market spillover indexes of 30.84% and 28.96%, respectively. Similarly, the four stock markets (EUROSTOXX50, FTSE100, NIKKEI225, and SP500) are also highly connected with one another. Note that the stock and commodity markets exhibit a small amount of connectedness with the cryptocurrency markets, with cross-market spillover indexes between cryptocurrencies and other markets ranging between 2.46 and 6.74% (see the first two rows and first two columns of Panel A). The FROM and TO connectedness indexes show that the four stock markets are the largest receivers and transmitters of shocks in the system. Finally, the NET connectedness index shows that cryptocurrencies and commodities are net shock receivers, whereas the four stock markets are net shock transmitters.

Panel B shows the connectedness among markets in terms of TBPV jumps. The total connectedness index for the TBPV jump is 67.87%. Overall, we have observed similar findings for the jump connectedness and realized volatility connectedness results. These observations suggest that both volatility and jumps are significant drivers of spillovers across the cryptocurrencies, commodities, and stock markets.

Panel C presents the connectedness among the realized skewness of the cryptocurrency, commodity, and stock markets, where the total connectedness index is 35% (the lowest across the four moments). The FROM connectedness indexes show that cryptocurrencies (BTC and ETH), EUROSTOXX 50, and FTSE100 are the main receivers of shocks, whereas the TO connectedness indexes show that ETH, EUROSTOXX50, and FTSE100 are the largest transmitters of shocks. The NET connectedness indexes show that Bitcoin (BTC) commodities are net shock receivers, whereas stock markets tend to be net shock transmitters.

Panel D shows the connectedness in realized kurtosis among the markets. The total connectedness index is 45.75%. The SandP 500 market receives the largest volume of shocks from the system (FROM connectedness index = 53.45%), followed by the FTSE100 (51.64%) and NIKKEI225 (51.56%). On the other hand, the SandP500, NIKKEI225, and FTSE100 indexes transmit the largest volume of shocks to the system, with TO connectedness indexes of 62.69%, 56.61%, and 47.13%, respectively. The NET connectedness indexes show that the EUROSTOXX50 and FTSE100 indexes are net shock receivers in kurtosis, while the other markets are net shock transmitters.

Overall, the results in Table 2 indicate that connectedness across cryptocurrencies, commodities, and stock markets is mixed across moments. The total connectedness is largest for realized volatility, followed by jumps, kurtosis, and skewness. Note that all connectedness indexes exceed 35%. This implies that, when compared with the extremely strong linkage in volatility and jumps, the markets are still significantly connected at higher moments (skewness and kurtosis). Thus, significant linkages among the markets still exist when low-probability events occur. However, the roles of the markets as shock transmitters and receivers vary across moments, which implies that markets may have different reactions under low-probability events (Zhang et al. 2022). Based on Table 2, the stock markets, and the SandP 500 index in particular, have the strongest spillover capacity, which is in line with the dominant role of the U.S. market in global financial markets. Moreover, own-market spillovers (the diagonal elements in Table 2) increase at higher moments, which means that each market is influenced by its internal shocks under low-probability events.

Tables 3 and 4 decompose the time-domain spillover indexes in Table 2 into short- and long-term horizons. Note that both frequency connectedness tables are estimated by the BK18 model with a 200-day window, a lag length of order 1 (BIC), and a 10-step-ahead forecast. We define the short term as a horizon of to 1–5 days (a trading week) and the long term as a horizon of 6 days and more. Overall, the tables show that most spillovers among the markets are concentrated in the short run, as the connectedness indexes are larger in Table 3 than in Table 4. Moreover, Tables 3 and 4 also show that each market’s individual shocks account for the largest share of its forecast error variance since the diagonal elements are the largest elements in all the connectedness matrices.

**Table 3:** Realized estimates connectedness table in the short-term horizon (1–5 days)

	BTC	ETH	EURO STOXX50	FTSE100	NIKKEI 225	SP500	Brent	Gold	FROM
*Panel A: realized volatility*									
BTC	24.37	19.24	3.77	3.59	3.71	3.67	2.28	2.61	38.87
ETH	19.61	23.61	3.35	3.69	4.08	4.1	2.28	2.64	39.75
EURO STOXX50	1.78	1.57	17.49	14.92	8.96	10.87	7.82	4.72	50.64
FTSE100	1.67	1.49	11.42	15.16	8.68	11.19	6.79	4.71	45.95
NIKKEI225	1.8	1.8	8.73	9.82	16.93	8.58	5.75	4.99	41.47
SP500	1.76	1.84	9.03	11.53	7.8	14.29	4.79	3.67	40.41
Brent	1.89	2.27	8.95	9.51	6.22	6.47	24.05	4.91	40.21
Gold	3	2.45	6.77	7.02	4.96	5.29	4.88	34.25	34.36
TO	31.5	30.65	52.02	60.07	44.41	50.17	34.59	28.25	331.66
Own	55.87	54.26	69.51	75.23	61.35	64.45	58.64	62.5	TCI
NET	− 7.37	− 9.1	1.38	14.12	2.94	9.75	− 5.63	− 6.11	41.46
*Panel B: TBPV jumps*									
BTC	26.06	20.5	4.09	3.71	4.16	4.07	2.56	3.36	42.44
ETH	20.7	24.53	4.01	3.75	4.17	4.52	2.2	3.67	43.01
EURO STOXX50	1.87	1.8	16.45	14.8	10.09	12.53	7.4	5.75	54.23
FTSE100	2.04	1.99	13.59	15.01	9.83	11.99	7.42	5.65	52.51
NIKKEI225	2.29	2.26	10.72	10.83	16.85	10.07	6.63	5.85	48.66
SP500	1.96	2.14	11.21	11.88	9.19	15.27	5.77	4.88	47.02
Brent	1.75	1.98	9.2	9.2	7.63	7.02	24.11	5.66	42.44
Gold	3.57	2.86	7.86	6.92	5.71	6.02	4.83	36.4	37.77
TO	34.17	33.54	60.68	61.08	50.77	56.22	36.81	34.81	368.08
Own	60.24	58.07	77.13	76.09	67.62	71.49	60.92	71.22	TCI
NET	− 8.27	− 9.47	6.44	8.57	2.12	9.2	− 5.63	− 2.96	46.01
*Panel C: realized skewness*									
BTC	63.24	21.62	0.82	1.1	0.54	1.01	0.77	0.74	26.61
ETH	21.24	62.81	1.03	1.23	0.83	1.19	0.27	0.86	26.64
EURO STOXX50	0.69	0.8	53.46	12.35	7.92	9.99	0.3	1.87	33.92
FTSE100	0.65	0.68	7.03	45.65	12	21.71	0.13	1.07	43.28
NIKKEI225	0.37	0.63	3.33	12.2	49.05	20.65	0.14	2.56	39.88
SP500	0.7	0.96	4.92	20.02	18.47	42.29	0.09	1.96	47.12
Brent	1.42	1.4	2.15	4.68	3.7	3.23	69.92	2.13	18.71
Gold	0.84	0.84	1.41	1.73	3.88	3.5	0.21	76.85	12.41
TO	25.91	26.93	20.7	53.31	47.34	61.28	1.91	11.19	248.55
Own	89.15	89.75	74.16	98.96	96.39	103.56	71.83	88.03	TCI
NET	− 0.7	0.29	− 13.23	10.04	7.46	14.16	− 16.8	− 1.22	31.07
*Panel D: realized kurtosis*									
BTC	50.52	22.9	2.55	2.51	3.18	1.49	2.05	2.44	37.13
ETH	23.16	48.42	2.41	3.29	4.01	2.31	2.1	2.88	40.17
EURO STOXX50	3.9	3.9	49.48	6.83	5.74	5.37	4.51	7.25	37.5
FTSE100	2.16	2.79	5.34	43.64	11.05	15.93	3.37	6.86	47.5
NIKKEI225	2.86	2.71	3.49	8.89	42.34	17.67	4.48	8.64	48.74
SP500	1.97	1.98	3.57	11.05	12.24	41.74	5.83	12.74	49.38
Brent	2.75	2.84	4.02	4.03	4.75	4.02	60.96	3.08	25.48
Gold	3.47	3.78	4.52	6.7	9.24	9.73	5.88	44	43.31
TO	40.27	40.9	25.9	43.3	50.21	56.51	28.22	43.89	329.2
Own	90.79	89.31	75.38	86.94	92.55	98.25	89.18	87.88	TCI
NET	3.14	0.73	− 11.6	− 4.2	1.47	7.13	2.74	0.58	41.15

See Table 2

**Table 4:** Realized estimates connectedness table in the long-term horizon (6-Infinity days)

	BTC	ETH	EURO STOXX50	FTSE100	NIKKEI225	SP500	Brent	Gold	FROM
*Panel A: realized volatility*									
BTC	12.11	11.6	2.21	2.72	3.55	2.38	0.87	1.32	24.66
ETH	9.35	14.05	1.8	2.43	4.28	2.64	0.98	1.1	22.59
EURO STOXX50	0.68	1	8.04	7.86	4.41	5.41	2.67	1.8	23.83
FTSE100	0.87	1.36	8.16	10.3	5.39	7.36	3.44	2.01	28.59
NIKKEI225	0.99	1.3	7.08	8.34	11.15	6.43	3.92	2.39	30.45
SP500	0.82	1.67	8.36	10.46	6.81	11.61	3.34	2.24	33.69
Brent	1.07	1.24	6.38	6.1	2.65	3.37	12.83	2.08	22.9
Gold	1.41	1.8	3.24	4.58	3.24	2.75	2.22	12.15	19.23
TO	15.19	19.97	37.23	42.5	30.32	30.34	17.44	12.94	205.94
Own	27.29	34.02	45.28	52.8	41.47	41.96	30.27	25.1	TCI
NET	− 9.47	− 2.62	13.4	13.91	− 0.12	− 3.34	− 5.46	− 6.29	25.74
*Panel B: TBPV jumps*									
BTC	11.36	11.08	1.43	1.69	2.56	1.58	0.95	0.84	20.13
ETH	9.39	14.45	1.14	1.21	2.9	1.79	0.88	0.7	18.01
EURO STOXX50	0.86	1.1	7.47	6.29	3.87	5.06	2.52	2.15	21.85
FTSE100	0.9	1.24	7.58	7.66	4.36	5.87	2.84	2.02	24.81
NIKKEI225	1.03	1.1	6.35	5.93	8.64	5.63	3.63	2.18	25.86
SP500	0.81	1.58	7.89	7.63	5.2	9.58	2.85	2.16	28.12
Brent	1.01	0.91	4.98	5.23	3.3	3.28	12.24	2.5	21.21
Gold	1.55	1.65	2.67	3.09	2.04	1.96	1.91	10.96	14.87
TO	15.55	18.68	32.04	31.08	24.22	25.17	15.58	12.56	174.87
Own	26.91	33.13	39.5	38.74	32.86	34.75	27.82	23.51	TCI
NET	− 4.58	0.67	10.18	6.26	− 1.64	− 2.96	− 5.63	− 2.31	21.86
*Panel C: realized skewness*									
BTC	7.02	2.69	0.08	0.05	0.05	0.1	0.13	0.05	3.14
ETH	2	8.14	0.1	0.08	0.08	0.09	0.02	0.05	2.4
EURO STOXX50	0.08	0.14	6.93	2.17	1.3	1.74	0.01	0.25	5.69
FTSE100	0.08	0.07	0.95	5.61	1.5	2.72	0.01	0.14	5.47
NIKKEI225	0.05	0.09	0.43	1.53	6.05	2.52	0.02	0.39	5.02
SP500	0.09	0.15	0.62	2.35	2.15	4.98	0.01	0.25	5.62
Brent	0.23	0.2	0.35	0.7	0.53	0.47	8.67	0.22	2.7
Gold	0.14	0.07	0.14	0.2	0.42	0.38	0.03	9.36	1.39
TO	2.67	3.41	2.67	7.08	6.03	8.01	0.21	1.35	31.42
Own	9.69	11.55	9.6	12.69	12.08	12.99	8.88	10.71	TCI
NET	− 0.47	1	− 3.02	1.61	1.01	2.4	− 2.49	− 0.04	3.93
*Panel D: realized kurtosis*									
BTC	6.7	3.41	0.37	0.27	0.26	0.43	0.39	0.51	5.65
ETH	2.33	6.06	0.53	0.61	0.48	0.15	0.42	0.84	5.36
EURO STOXX50	0.51	0.36	7.14	1.08	1.09	1.1	0.58	1.17	5.88
FTSE100	0.33	0.29	0.38	4.72	0.97	1.61	0.16	0.4	4.14
NIKKEI225	0.29	0.11	0.13	0.19	6.1	1.22	0.29	0.6	2.82
SP500	0.17	0.19	0.2	0.78	1.51	4.81	0.67	0.56	4.07
Brent	0.35	0.29	1.22	0.44	0.76	0.31	9.48	0.72	4.09
Gold	0.48	0.37	0.42	0.47	1.32	1.35	0.36	7.92	4.78
TO	4.46	5	3.25	3.83	6.39	6.18	2.88	4.79	36.78
Own	11.16	11.06	10.4	8.55	12.49	10.99	12.36	12.71	TCI
NET	− 1.19	− 0.36	− 2.62	− 0.31	3.57	2.11	− 1.21	0.02	4.60

See Table 2

Panel A of Tables 3 and 4 presents the frequency connectedness in volatility. The short-term total connectedness index is 41.46%, while the long-term total connectedness index is 25.74%. This suggests that most shock spillovers occur over the short run; however, significant amounts of shocks persist through over the long run as well. In both the short and long run, the four stock indexes receive and transfer the largest amount of shocks to the system, which is indicated by their large FROM and TO connectedness. On the other hand, gold is the least connected to other markets, since its FROM and TO connectedness indexes are the smallest among all the markets across both the short and long run. In the short run, the four stock indexes are net transmitters of shocks, with the FTSE 100 and SandP 500 being the largest net shock transmitters (NET connectedness indexes = 14.12 and 9.75%, respectively). By contrast, cryptocurrency and commodity markets are net shock receivers over the short run. Interestingly, the NIKKEI 225 and SandP 500 indexes become net shock receivers over the long run, indicating a change in the dynamic relationship across the markets between the short and long run.

Panel B of Tables 3 and 4 presents the frequency connectedness in jumps. The short-term total connectedness index is 46.01%, while the long-term total connectedness index is 21.86%. In the short run, the largest receiver of jump shocks in the short run is the EUROSTOXX 50 and the FTSE100 indexes (FROM connectedness = 54.23% and 52.51%, respectively), and the smallest receiver of jump shocks in the short run is the gold index (FROM connectedness = 37.77%). The four stock indexes contribute the largest amount of shocks to the system in the short run (TO connectedness greater than 50%). In the short run, cryptocurrencies and commodities are net shock receivers (negative NET connectedness), while stock markets are net shock transmitters (positive NET connectedness). Similar conclusions can be drawn for long-run jump connectedness; however, we note that all connectedness indexes are smaller in the long run. This indicates the dissipation of the jump shocks in the long run. However, our results show that the total connectedness index is still greater than 20%; thus, a significant amount of jump shock spillovers persist in the long run. Interestingly, the roles of markets as net shock transmitters and receivers switches between the short and long run. For example, Ethereum (ETH) is a net shock receiver in the short run, but a net shock transmitter in the long run. The NIKKEI225 and SP500 indexes are net shock transmitters in the short run but net shock receivers in the long run.

Panel C of Tables 3 and 4 presents the short- and long-term skewness connectedness indexes. We find that the skewness connectedness indexes dissipate rapidly. Specifically, the total connectedness index is 31.07% in the short run, which is significantly reduced to 3.93% in the long run. This suggests that markets quickly stabilize following a shock in skewness. The EUROSTOXX50 and FTSE100 indexes receive the largest amount of shocks from the system and also transfer the largest amount of shocks to the system, both in the short and long run. The two commodities (Brent crude oil and gold) receive the smallest volume of shocks from the system and transfer the smallest volume of shocks to the system. In contrast to the connectedness in volatility and jumps, we do not observe a switch in the net receiver/transmitter properties of the markets in either the short or long run. This suggests that skewness connectedness follows a more predictable pattern than jumps and volatility connectedness.

Panel D of Tables 3 and 4 presents kurtosis connectedness indexes. The short-run total connectedness index is 41.15%, which reduces to 4.60% in the long run. This sharp decline in total kurtosis connectedness indicates the fast processing of information among markets during low-probability events; thus, most spillovers are concentrated in the short run. In the short run, the SandP 500 receives and transmits the largest volume of shocks from and to the system (FROM connectedness = 49.38%, TO connectedness = 56.51%). Brent crude oil receives and transmits the smallest volume of shocks from and to the system (FROM connectedness = 25.48%, TO connectedness = 28.22%). We observe several changes in the net connectedness between the short and long run. Specifically, cryptocurrencies (BTC and ETH) and Brent crude oil are net shock transmitters in the short run, but net shock receivers in the long run. Interestingly, gold remains a net kurtosis transmitter across both the short and long run, while it serves as a net receiver of shocks in other moments (volatility, jumps, and skewness). This is in line with investors’ flight-to-quality behavior under the occurrence of low-probability events, as gold is typically considered a safe-haven asset.

In summary, our static connectedness results show various spillover patterns among cryptocurrencies, commodities, and stock markets across the moments. This highlights the relevance of analyzing market spillovers at higher-order moments. We find that volatility and jump connectedness are the largest; they, thus, contain more information about the markets than of other moments (skewness and kurtosis). Moreover, significant volatility and jump connectedness persist across the long run, suggesting that shocks in volatility and jumps are transmitted for longer periods. In contrast, skewness and kurtosis connectedness are mostly concentrated in the short run, which suggests a faster processing of information among markets in response to low-probability events. Our results also indicate that market-specific shocks are the largest contributors to the forecast error variance across all moments and frequencies. Finally, we can also identify clusters of markets that are highly connected to one another from the connectedness tables. Specifically, the four stock markets and two cryptocurrencies form their own individual clusters, with high connectedness within the clusters and smaller connectedness outside the clusters. These are expected, as these clusters represent similar investment options governed by similar shocks. Commodities (Brent crude oil and gold) are the least connected to other markets.

### Dynamic total spillover analysis

**Fig. 2:**
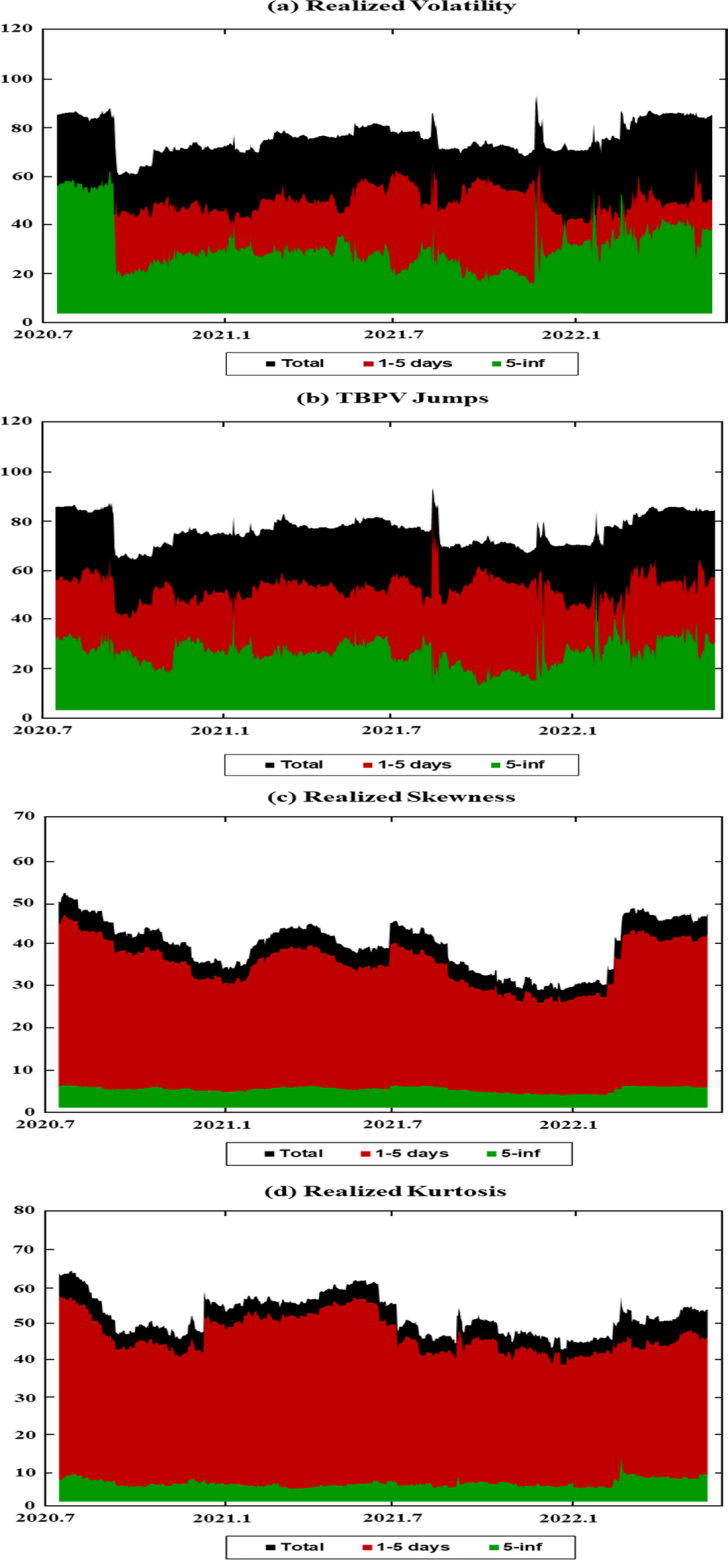
Dynamics of total spillover indexes. *Notes* Results are based on a VAR model with lag length of order one (AIC) with a 10-step-ahead generalized forecast error variance decomposition and a rolling window of 200 days. Black shaded areas illustrate the total connectedness index (TCI) estimated by the DY12 model while the brown and green-shaded areas represent the shot-term (1–5 days) and long-term (6 days-infinite days), respectively

In this section, we present the results of the dynamic spillovers among cryptocurrencies, commodities, and stock markets across the moments. Following Diebold and Yilmaz (2012), we estimate the dynamics of spillover indexes using a rolling window of 200 days (corresponding to a trading year).^9^ Figure 2 presents the time-varying total spillover indexes among the markets for realized volatilities, jumps, skewness, and kurtosis. This figure shows that spillovers vary over time and increase during crises.

Figure 2A shows that the realized volatility spillover ranges between 60 and 90%, and attains the highest level in 2020 and at the beginning of 2022. These periods correspond to the COVID-19 pandemic and the onset of the Russo-Ukrainian War; thus, the change in the spillover patterns reflects the reactions of markets to these financial and political events. Our results also show that short-term volatility connectedness tends to be greater than long-term volatility connectedness, except during the first year of the COVID-19 pandemic in 2020. These results suggest a higher level of shock persistence among markets at the beginning of the pandemic (Adekoya 2022; Vera-Valdés 2022; Carporale et al. 2022; Bentes 2021; Youssef et al. 2021; Shahzad et al. 2021a).

Figure 2B presents the dynamic spillover index for jumps, which fluctuate between 60 and 90%. The index also peaks in 2020, which corresponds to the COVID-19 pandemic, and again at the beginning of 2022, which corresponds to the onset of the Russo-Ukrainian War. This figure also indicates the dominance of connectedness in the short run compared to that of the long run. Altogether, the results in Fig. 2A, B suggest a high degree of volatility and jump connectedness across markets, which intensifies during crisis periods. Note that while short-run connectedness tends to be larger in Fig. 2A, B, a significant amount of connectedness (> 20%) persists across the long run. This suggests that volatility and jump shocks are transmitted over longer periods.

Figure 2C shows the dynamic spillover index for skewness. The skewness connectedness index ranges between 35 and 50% and tends to be lower than the volatility and jump connectedness index. As shown in Fig. 2C, we have observed an increase in skewness connectedness at the beginning of the sample (the first phase of the COVID-19 pandemic), in mid-2021 in response to the increasing policy uncertainty in cryptocurrency markets, and in early 2022 in response to the Russo-Ukrainian War. However, most skewness spillovers are concentrated in the short run and dissipate quickly in the long run.

Figure 2D shows the kurtosis total spillover index across markets. The kurtosis connectedness index tends to be lower and less volatile than the spillover indexes at other moments. Of note is out observation of a sharp increase in kurtosis connectedness during the first phase of the COVID-19 pandemic and at the beginning of the Russo-Ukrainian War in 2022. Moreover, most of the connectedness in kurtosis occurs in the short run, implying that shocks tend to dissipate quickly with respect to kurtosis spillovers.

Overall, Fig. 2 shows strong connectedness among cryptocurrencies, commodities, and stock markets, with volatility connectedness exhibiting a stronger and more variable pattern than connectedness at other moments. Moreover, short-run spillovers tend to be larger than long-run spillovers, indicating that these markets are more connected in the short run than in the long run. However, we note that while kurtosis spillovers dissipate rather quickly (as indicated by their long-run connectedness of less than 10%), the spillovers at other moments (volatility, jumps, and skewness) dissipate more slowly. At the same time, given that volatility and jump spillovers are higher than those of the other moments, both in the long and short run, our results show that volatility and jumps can provide more information about the markets than other higher-order moments. Figure 2 also illustrates the impact of adverse events, such as the COVID-19 pandemic and the Russo-Ukrainian War, on increasing the spillovers among the markets across all moments. These results are comparable to those of previous studies that show higher spillovers in higher-order moments across markets during periods of high volatility (Zhang et al 2022; Finta and Aboura 2020).

### Dynamic net connectedness analysis

**Fig. 3:**
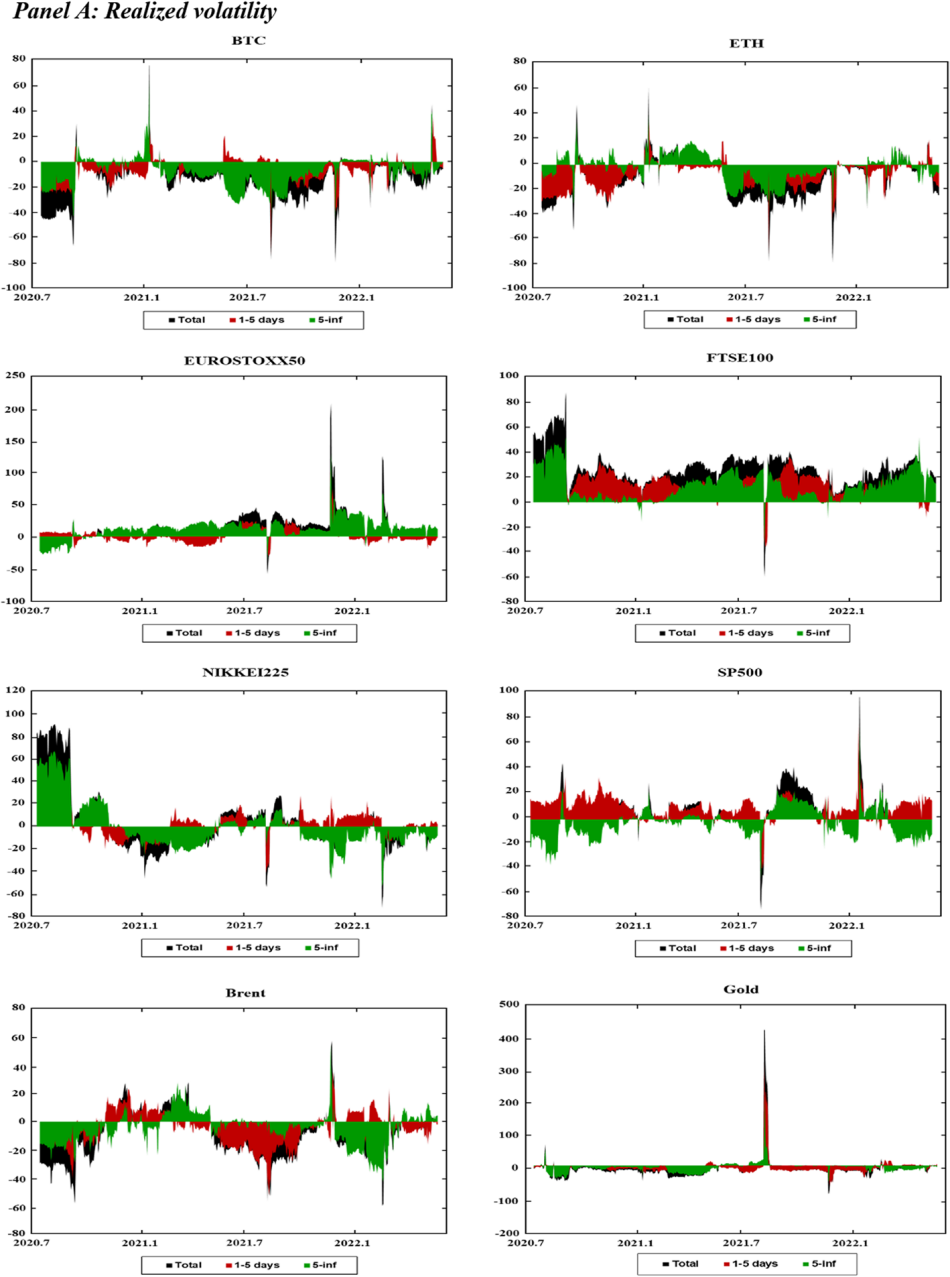

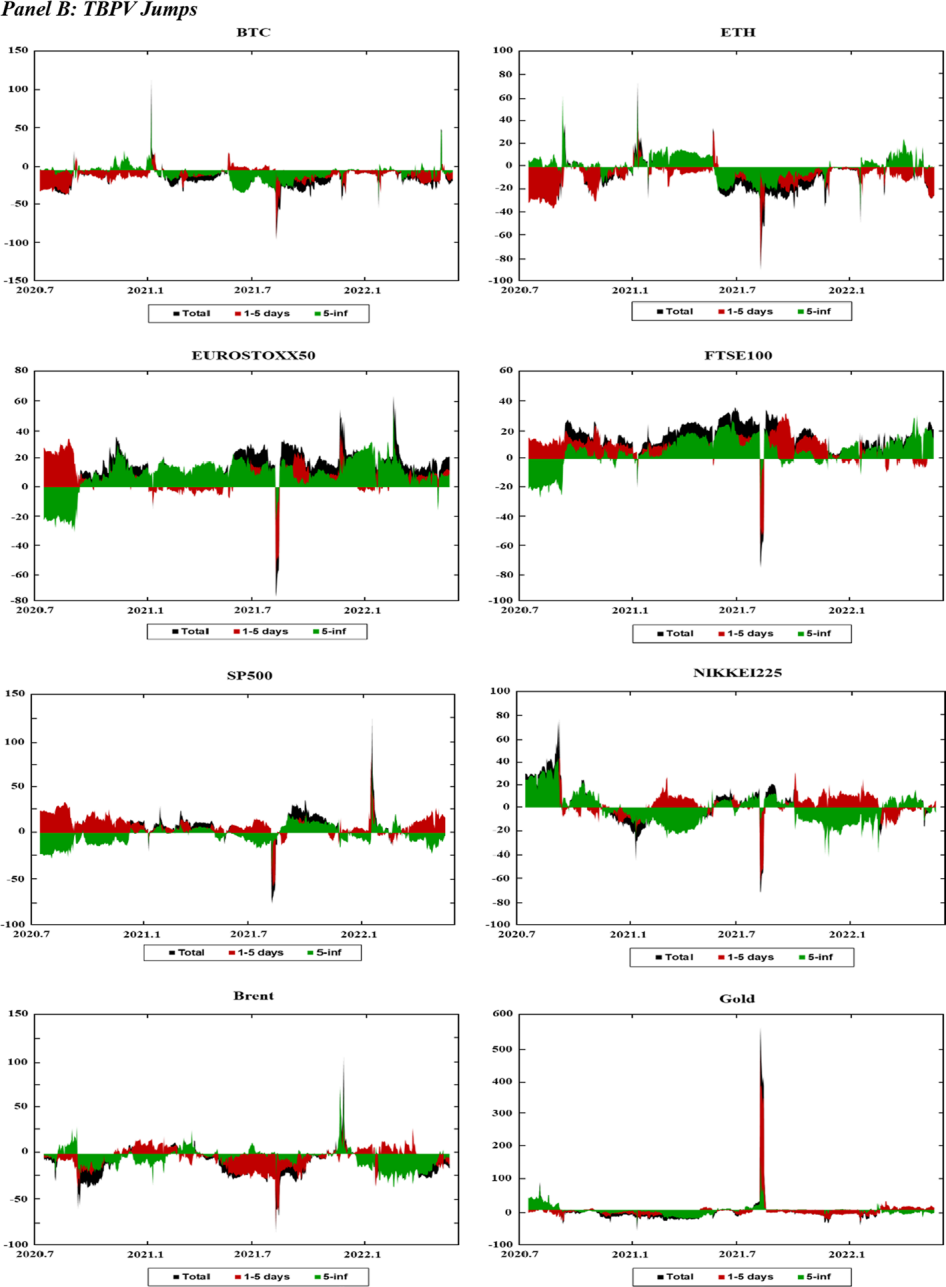

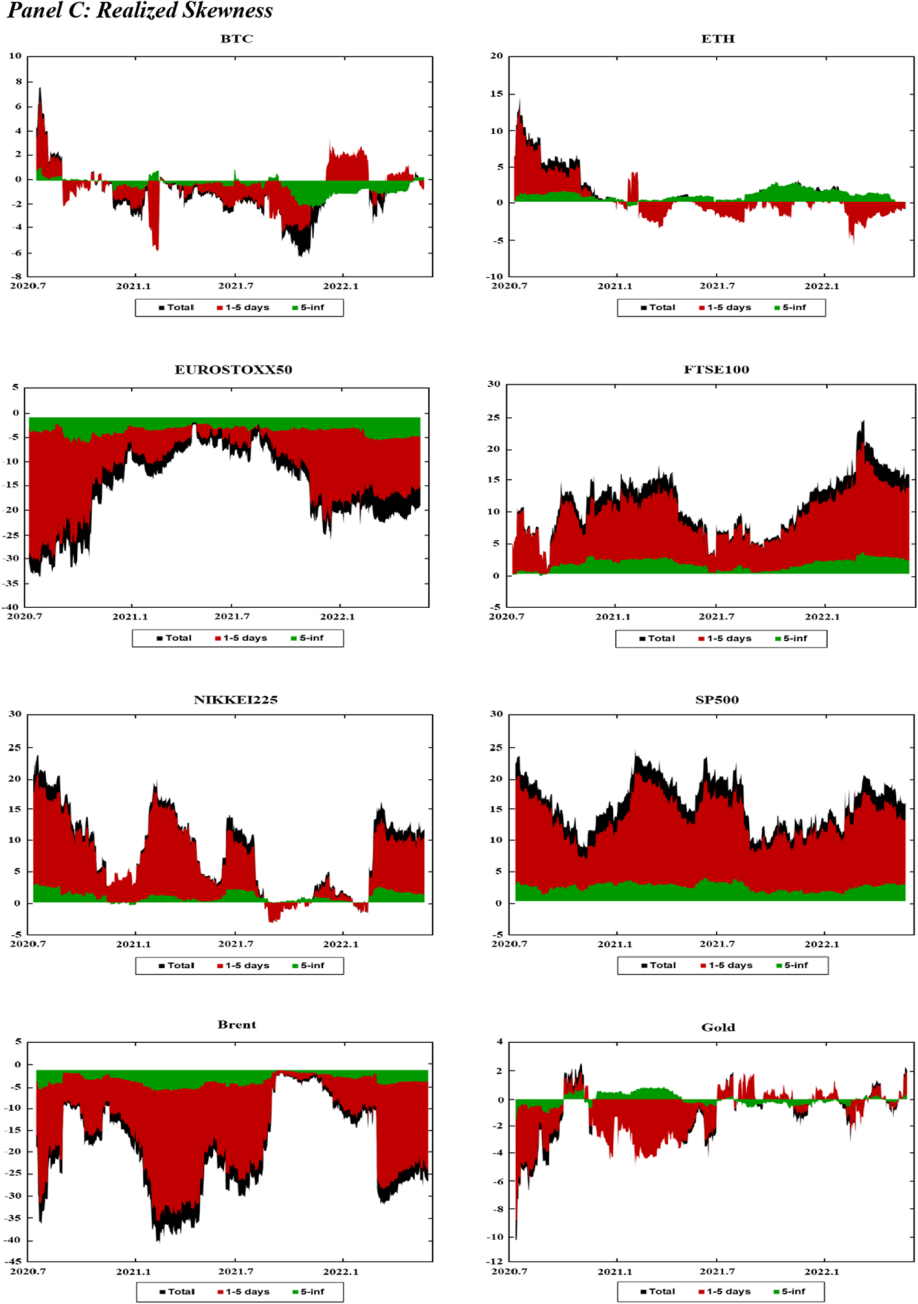

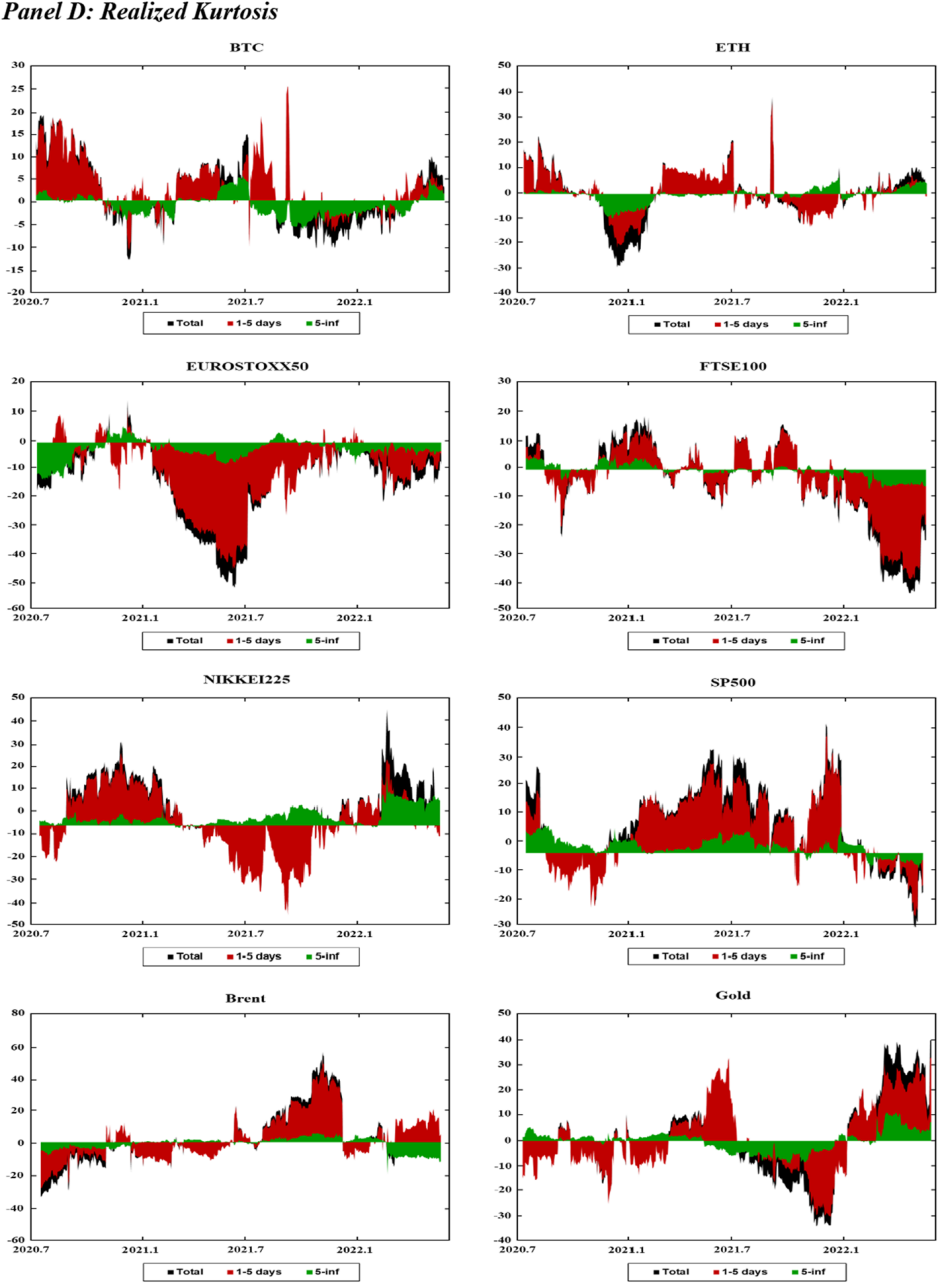
Dynamics of Time–frequency net connectedness. *Notes* This figure displays the time–frequency dynamics of the net directional connectedness across the cryptocurrency, stock, and commodity markets using the DY12 and BK18 methods. The net directional connectedness measures are the difference between directional “TO” spillovers and directional “FROM” spillovers. Positive (negative) connectedness values indicate that the corresponding variable is a net transmitter (receiver) of connectedness to (from) all the other variables. The black-shaded area indicates net connectedness index. The brown shaded area represents the net connectedness index over the short-term horizon (1–5 days). The green-shaded area reflects the net connectedness index over the long-term horizon (6-inf. days)

Figure 3 presents the dynamic net connectedness indexes for the markets across moments (realized volatility, jumps, skewness, and kurtosis). This figure presents useful information on the role of each market as a net shock transmitter and receiver across both the short and long run.

Figure 3A shows the net volatility spillovers in the markets over time. In Fig. 3A, BTC, ETH, and Brent tend to be net shock receivers in both the short and long run throughout the sampling period. Gold is a shock receiver throughout most of the sampling period, except at the beginning of 2020, mid-2021, and again at the beginning of 2022. These periods are characterized by extreme events that increase the volatility of the markets; for example, the initial phase of the COVID-19 pandemic, the Chinese ban on cryptocurrency, and the onset of the Russo-Ukrainian War. As gold is considered a safe-haven investment, many investors fly to safety during these periods, thereby increasing the role of gold as a shock transmitter in the system. The four stock indexes exhibit fluctuating patterns throughout the sampling period. For example, the FTSE100 and NIKKEI225 indexes are net shock transmitters at the beginning of the sampling period, whereas the EUROSTOXX50 and SP500 indexes are net shock receivers. In subsequent periods, while the FTSE100 index remains the net shock transmitter, other stock indexes fluctuate between being a shock transmitter and receiver.

Figure 3B presents the net jump spillover indexes, which show patterns similar to the net volatility spillover indexes. However, the net jump spillover indexes tend to be closer to zero than that of the net volatility spillover indexes. This indicates a higher balance between the number of shocks that each market receives from, and transmits to, the system under jump spillovers.

Figure 3C shows the net realized skewness connectedness indexes. The net spillover indexes for the stock indexes (EUROSTOXX50, SP500, FTSE100, and NIKKEI225) experienced substantial changes during the initial phase of the COVID-19 pandemic (2020) and at the beginning of the Russo-Ukrainian War. In addition, these changes tend to be concentrated across the short run, and stabilizing over the long run. This is indicated by the fact that the long-run net connectedness for these markets is close to zero compared to the short-run net connectedness. Figure 3C shows that the EUROSTOXX50 is a net shock receiver in terms of skewness, whereas the other stocks are net shock transmitters. The Brent crude oil market experiences a large change in net spillovers at the beginning of 2020, in mid-2021, and in early 2022 in response to events such as the Russia-Saudi Arabia oil price war (2020), rising oil demand associated with increasing post-pandemic travel (2021), and the Russo-Ukrainian War (2022). Similar to stock markets, changes in the net connectedness of crude oil tend to be more concentrated in the short run and stabilize over the long run. Figure 3C shows that the skewness net spillovers are more stable for other markets such as Bitcoin (BTC), Ethereum (ETH) and gold, as the net spillover indexes for these markets tend to be close to zero throughout the sampling period.

Figure 3D shows the net realized kurtosis connectedness indexes for these markets. Compared to the net connectedness indices at other moments, the net kurtosis connectedness indexes fluctuate between positive and negative values throughout the sampling period. This indicates weak predictability of net connectedness among markets during low-probability events. Similar to the results at other moments, we also observe substantial changes in the net kurtosis connectedness at the beginning of 2020 and 2022. Moreover, the net kurtosis connectedness is more stable (close to zero) in the long run than in the short run.

Overall, the net connectedness analysis shows a change in the connectedness patterns under significant market events. However, most of the changes are observed in the short run, while the long-run net connectedness tends to be closer to zero. These results suggest that while markets respond to adverse events in the short run, they gradually stabilize towards long-run behavior over time. They are also consistent with previous evidence of a regime switch in spillover across markets during crisis events such as the COVID-19 pandemic (Hui and Chan 2022; Shahzad et al. 2021a; Shahzad et al. 2021b; Yousaf and Ali 2020).

### Connectedness network analysis

**Fig. 4:**
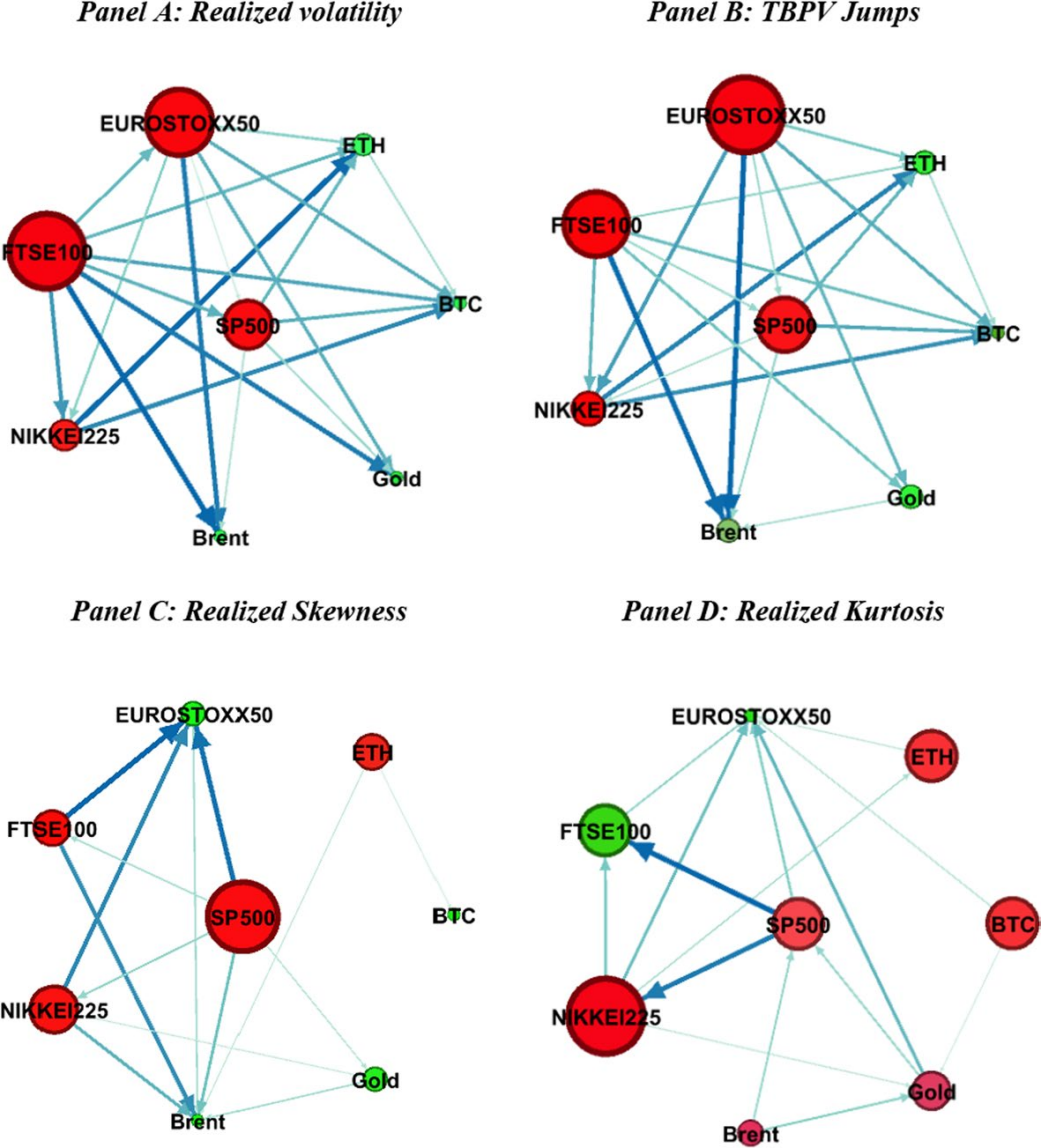
Connectedness network in the time domain. *Notes* This figure presents the network of connectedness based on the DY12 method. Red (green) nodes illustrate the net transmitter (receiver) of shocks. Vertices are weighted by averaged net pair-wise directional connectedness measures. The size of nodes represents weighted average net total directional connectedness. The thickness of the edge (blue line) indicates the strength of connectedness, and the arrow of the edge expresses the direction of connectedness

**Fig. 5:**
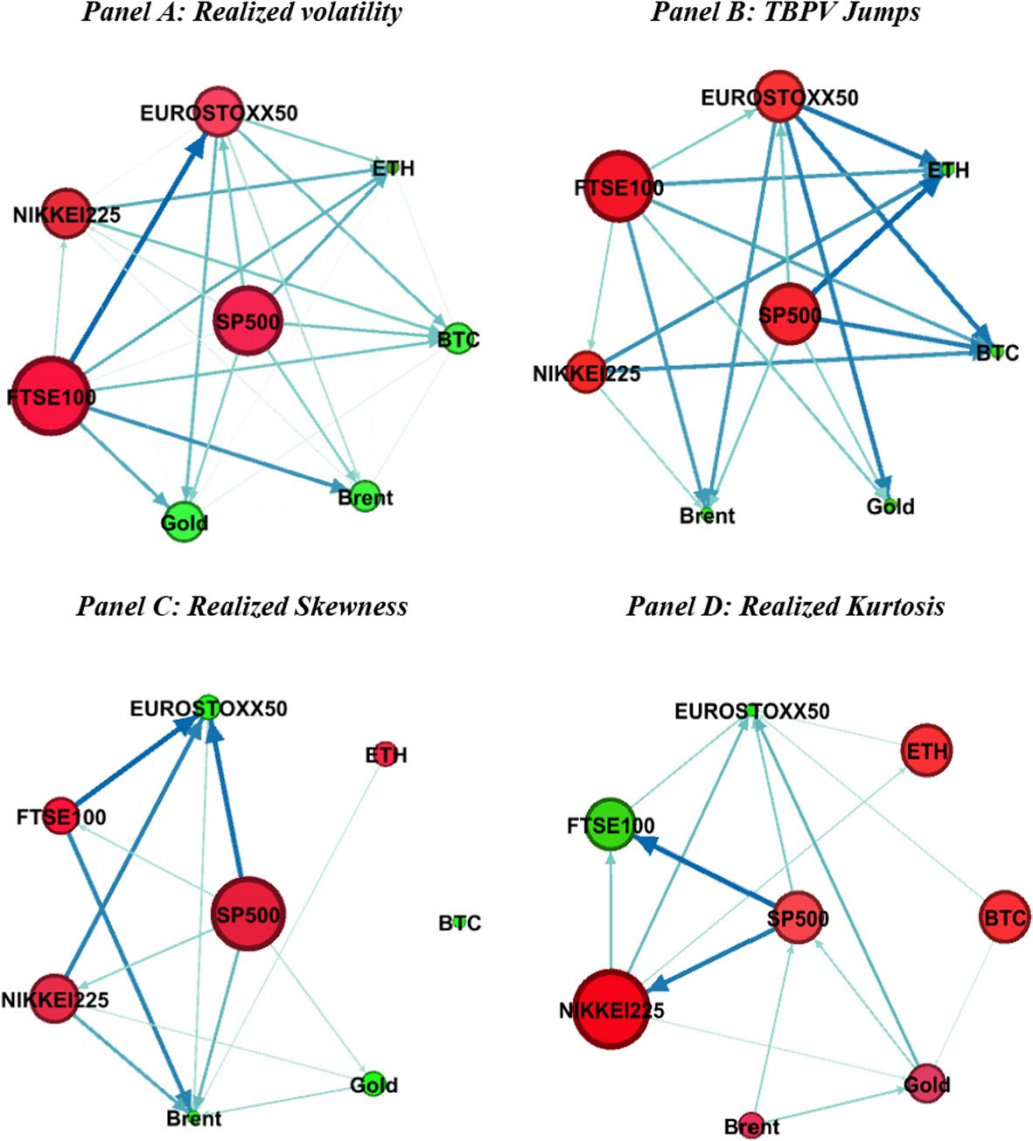
Connectedness network in the short-term horizon. *Note* This figure presents the network of short-term connectedness based on the BK18 method. See Fig. 4

**Fig. 6:**
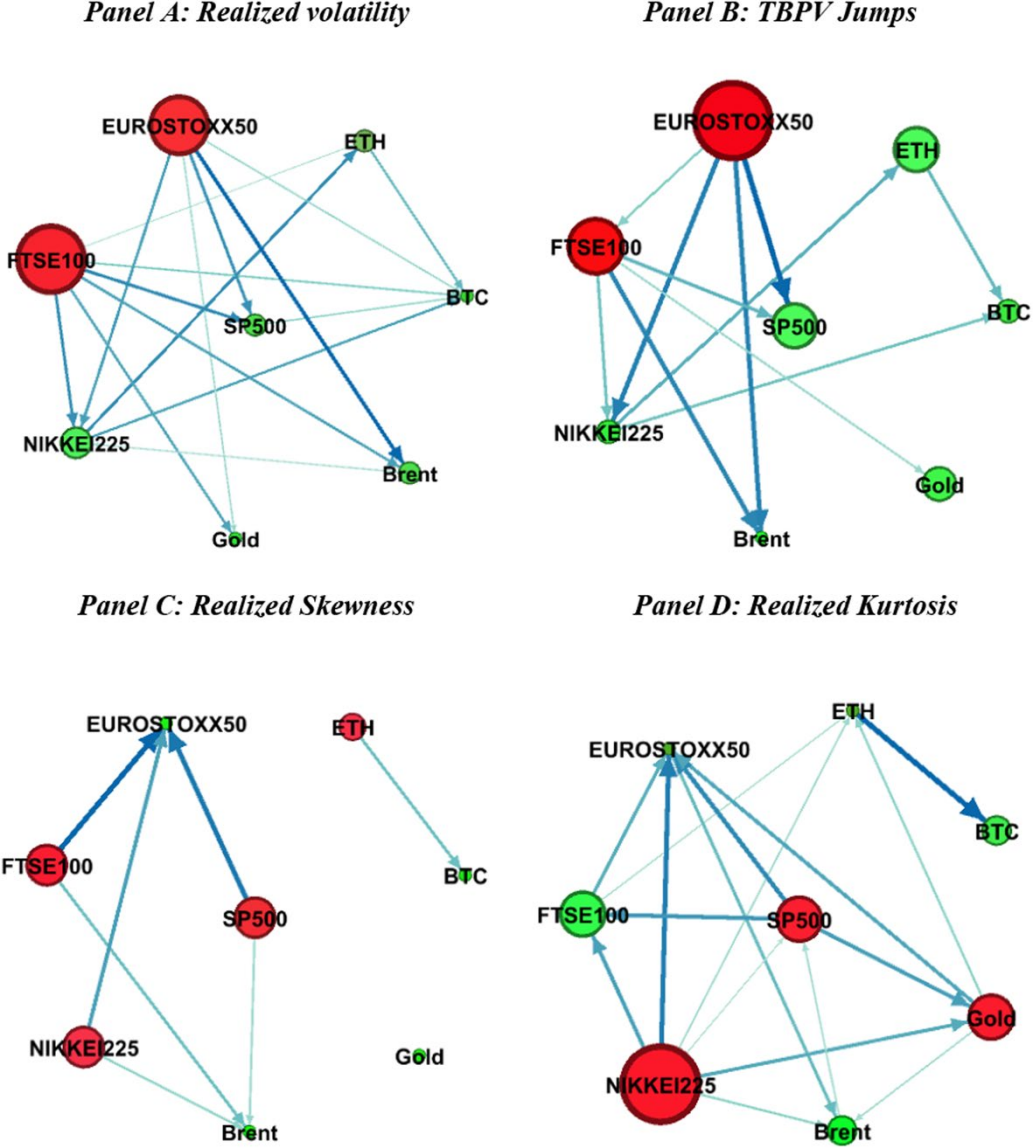
Connectedness network in the long-term horizon. *Note* This figure presents the network of long-term connectedness based on the BK18 method. See Fig. 4

Next, we have visualized our analysis presented in the previous sections using network graphs. Figures 4, 5 and 6 present the network connectedness graphs in the time domain, the short run, and the long run frequencies, respectively. The blue (yellow) nodes illustrate the net transmitter (receiver) of shocks. Vertices were weighted using averaged net pairwise directional connectedness measures. The size of the nodes represents weighted average net total directional connectedness. The thickness of the edge (gray line) indicates the strength of connectedness, and the arrow of the edge expresses the direction of connectedness. In contrast to previous analyses that focused on the relationship between each market and the entire system, network analyses focus on the directional relationships between each pair of markets.

Figure 4 presents network connectedness graphs in the time domain. In Fig. 4, the SP500 and NIKKEI225 indexes are the net shock transmitters across all moments. The FTSE100 index is a net shock transmitter in volatility, jumps, and skewness, but a net shock receiver in kurtosis. The EUROSTOXX50 index is a net shock transmitter in volatility and jumps but a net shock receiver in skewness and kurtosis. Brent crude oil and gold are net shock receivers for volatility, jumps, and skewness, but net shock transmitters for kurtosis. Finally, the two cryptocurrencies are net shock receivers in volatility and jumps, but their roles switch at other times. Figure 4 also shows that the markets are highly linked in volatilities and jumps; however, their pairwise directional spillovers weaken when skewness and kurtosis are considered. Stock markets are highly connected to one another across all moments, while commodity and cryptocurrency markets tend to decouple from the system in terms of skewness and kurtosis. Altogether, these results suggest a higher information content of volatility and jumps in spillover patterns across markets compared to skewness and kurtosis. However, an analysis of skewness and kurtosis spillovers is still important as it reveals useful insights into spillovers across markets under low-probability events.

Figure 5 presents the network connectedness graphs in the short run, whereas Fig. 6 shows the graphs for the long run. We find that the roles of the markets as net shock transmitters and receivers in the short run are similar to the patterns in the time-domain graphs in Fig. 4. This indicates a significant contribution of short-run spillover patterns to general spillovers across markets. Moreover, the strength of the pairwise directional connectedness weakens in the long run. These results are consistent with our previous findings that shock transmission across cryptocurrency, commodity, and stock markets is concentrated in the short run and dissipates in the long run.

### Discussion of the results

In summary, our estimation results show that the cryptocurrency markets are highly connected to the stock and commodity markets during the period 2020–2022, where the average total connectedness indexes are larger than 35% for all moments. We also find a significant impact of adverse events such as the COVID-19 pandemic financial crisis and the Russo-Ukrainian War on spillovers across markets. The high spillover effects among cryptocurrencies, stocks, and commodities during our sampling period of 2020–2022 are consistent with previous empirical studies that document significant contagion across financial markets since the start of the COVID-19 pandemic in 2020 (Ghosh et al. 2022; Carporale et al. 2022; Vera-Valdés 2022; Adekoya 2022; Hui and Chan 2022; Shahzad et al. 2021a; Shahzad et al. 2021b; Bentes 2021; Youssef et al. 2021; Yousaf and Ali 2020). Our study contributes to the extant body of literature on the matter by documenting the relationship between cryptocurrencies and other financial markets at higher-order moments. By doing this, we are able to capture the unique characteristics of financial returns—such as asymmetry, skewness, and fat tails—which could help investors account for asymmetric or fat-tail risks related to extreme market events (Zhang et al 2022; Finta and Aboura 2020). Our results show that the markets are still significantly connected under low-probability events such as downside risks or tail risks. In addition, the roles of markets as shock transmitters and receivers vary across moments, which highlights the relevance of analyzing spillovers at higher-order moments.

Our results also show that cross-market spillovers are stronger in the short term than in the long term. This implies that information processes quickly across the cryptocurrency, commodity, and stock markets; therefore, most shock spillovers are concentrated in the short run. Our results are consistent with those of previous studies (Qarni and Gulzar 2021; Pham 2021; Wang et al. 2021; Zhang and Wang 2022; Bhanja et al. 2023). However, while these studies document return spillovers, we analyze market interdependence across higher moments, thereby identifying important market behaviors during episodes of significant tail or crash risks. Compared with other moments, shocks in volatility and jumps are transmitted for longer periods and tend to persist in the long run. In addition, total connectedness is the largest for volatility and jumps, followed by kurtosis and skewness. Therefore, the second-order moment and its jump component are the largest contributors to the spillover patterns across commodities, cryptocurrencies, and stock markets. This result is consistent with the findings of Finta and Aboura (2020), who document the prominent role of volatility risk premium in spillovers among the U.S., U.K., German, and Japanese stock markets. Our result further corroborates several previous studies that find that the realized volatility spillover is stronger than the spillovers in skewness and kurtosis (Cui and Maghyereh 2022; Hasan et al 2021; Bouri et al 2021d; Yi et al. 2018).

## Conclusion and policy implications

This study analyzes the different higher moments through which cryptocurrencies, commodities, and equities can be related. Specifically, we consider spillovers across these markets in realized higher moments (volatility, skewness, and kurtosis) and in the jump components of volatility. Our results support the notion that higher moments and jumps in volatility contain important information about the cross-market spillovers between cryptocurrencies, commodities, and equities, which is relevant for effective portfolio management.

Using high-frequency (5-min interval) data between 2020 and 2022, we first calculate the realized higher-order moments of cryptocurrencies, stocks, and commodities. Next, we use the connectedness framework of DY12 and BK18 to analyze spillover patterns across markets and time horizons. Our results show varying degrees of spillovers across short- and long-run investment horizons. First, by using the time domain connectedness approach of DY12, we find that the markets are highly connected across all moments throughout the sampling period, with average total connectedness indexes exceeding 35%. The total connectedness is largest for volatility and jumps, followed by kurtosis and skewness. This implies that the second-order moment and its jump component are the largest contributors to the spillover patterns across commodities, cryptocurrencies, and stock markets. However, an analysis of higher-order moments is still relevant because it contains useful information about market characteristics under low-probability events. Our results show that the markets act as shock transmitters, and that receivers vary between volatility and higher-order moments; therefore, markets may have different reactions under low-probability events. Moreover, each market is influenced by its internal shocks under low-probability events, as own-market spillovers tend to increase under skewness and kurtosis spillover networks compared to the volatility spillover network.

In addition to analyzing the time-domain spillovers among the markets at various moments using the BK18 connectedness approach, our study also sheds light on their frequency spillovers, thereby identifying whether shocks are mostly transmitted in the short or long run. Our results show stronger short-run shock spillovers for all moments. However, significant volatility and jump connectedness persist in the long run; therefore, shocks in volatility and jumps are transmitted over longer periods. However, skewness and kurtosis connectedness are mostly concentrated in the short run and dissipate quickly in the long run. Next, we apply rolling window analysis to the DY12 and BK18 models so as to analyze how connectedness across markets varies over time. We find a significant impact of adverse events such as the COVID-19 financial crisis and the Russo-Ukrainian War on increasing spillovers across markets. These effects are significant across all moments and time horizons, reflecting changes in investors’ expectations and behaviors under extreme events.

Finally, our analysis reveals clusters of markets that are highly connected to one another. Specifically, the four stock markets and two cryptocurrencies form their individual clusters, with high connectedness within the clusters and smaller connectedness outside the clusters. By contrast, commodities (Brent crude oil and gold) are the least connected to other markets.

These results have important implications for portfolio management. First, because stock and cryptocurrency markets exhibit larger spillovers to the system, they can be a source of instability in strategic commodities such as crude oil or gold, which are usually considered hedging or safe-haven assets. Second, an econometric understanding of volatility and jump behavior across markets is relevant for asset management in both the short and long run because volatility and jump connectedness are the highest among the moments and persist across the long run. Third, an analysis of skewness and kurtosis spillovers can provide useful information on markets under low-probability events, particularly in the short run. Fourth, the surge of spillovers in higher-order moments during turbulent periods highlights the importance of understanding the behavior of market jumps and higher-order moment characteristics, in addition to considering the spillovers in returns under extreme market movements. Fifth, our results show the potential of commodities (Brent crude oil and gold) as hedging or safe-haven investments for cryptocurrencies and equities, especially in the long run; however, their hedging and safe-haven properties may weaken during crisis periods. Therefore, active portfolio management is essential during extreme events. Finally, policymakers who wish to stabilize cryptocurrency markets should pay attention to both volatility and crash risk (skewness and kurtosis) spillovers, as assets tend to exhibit different behaviors across moments, and especially during crises.

While our study provides informative empirical conclusions on the spillovers in higher-order moments between cryptocurrencies and other markets, several limitations are worth highlighting. First, we focused on Bitcoin and Ethereum, the two largest cryptocurrencies, while excluding other cryptocurrencies. Second, our sampling period captures the most recent dynamics of the cryptocurrency market; however, it is not long enough to capture the impacts of major events before 2019—such as the 2015–2016 oil gluts, the 2018 US-China trade war, and the 2018 cryptocurrency bubble crash. This study provides ample opportunities for future research. For example, future studies could investigate higher-moment connectedness among a wider range of cryptocurrencies and financial assets using other methodologies, such as Granger causality, quantile regressions, or time-varying parameter models. In addition, studies that analyze the impact of macroeconomic and financial factors on the interdependence between cryptocurrencies and broader financial markets can have important implications for investors and policymakers. Finally, studies assessing the effectiveness of cryptocurrency regulations would provide useful insights for society to fully reap the benefits of cryptocurrencies.

## Data Availability

The datasets generated and/or analysed during the current study are not publicly available due to data security but are available from the corresponding author on reasonable request.

## Abbreviations

BK18: Frequency-domain spillover index of Baruník and Křehlík (2018)
BTC: Bitcoin
BRENT: Brent crude oil
DY12: Diebold and Yilmaz (2012) spillover approach
ETH: Ethereum
GOLD: Gold
FEVD: Forecast error variance decompositions
KPPS: Koop et al. (1996)
TBPV: Threshold bi-power variation
VAR: Vector autoregressive model

## Footnotes

1 Fears of economic meltdown and inflationary pressure on the U.S. dollar at the beginning the COVID-19 pandemic pushed cryptocurrency prices. For example, by December 2020, Bitcoin prices had more than tripled since January 2020 and ended the year with a price of about $29,000, the highest it had ever been. Bitcoin doubled its value in the first half of 2021, then quickly fell back below $30,000 over the summer. In November 2021, Bitcoin hit another all-time high of over $68,000, before quickly dropping below $35,000 in January 2022. Since March 2022, Bitcoin price has trended downward. See more at https://time.com/nextadvisor/investing/cryptocurrency/bitcoin-price-history/.

2 For example, see Khin et al. (2017), Barunik et al. (2015, 2017, 2018), Khin et al. (2022).

3 Our rationale for studying the U.S., U.K., Eurozone, and Japan stock market is that these markets are the largest and most influential financial markets. In recent years, empirical evidence has shown that the interconnectedness between cryptocurrencies and stock markets have increased, particularly during periods of financial distress such as the period after 2020 (Zeng et al. 2020; Colon and McGee 2020; Maniff et al. 2020). In addition, we include oil prices in our model, because the high energy intensity in cryptocurrency mining, particularly Bitcoin mining, implies the interdependence between energy prices (such as oil) and cryptocurrency prices. Finally, we include gold prices in our empirical analysis, because of its safe-haven property. Our goal is to investigate the usefulness of gold in cryptocurrency portfolio management during the period after 2020.

4 Specifically, the connectedness framework of Diebold and Yilmaz (2012) captures the spillovers across markets in the time domain. Baruník and Křehlík (2018) later extend this framework to capture the spillovers across markets in the frequency domain, which allows researchers to identify the relationship among variables over different investment horizons (i.e. the short v. long run). These approaches have been applied widely in previous studies of financial market spillovers, for example, Ferrer et al. (2018), Reboredo et al. (2020), Zhang and Hamori (2021), Le et al. (2021), Adekoya et al (2021), Pham (2021), Naeem et al. (2022), Su et al. (2022), Dai et al. (2022). A static estimation of the connectedness models using data for the entire sampling period allows researchers to identify the average spillovers across all variables. In addition, previous research also relies on a rolling-window estimate of the connectedness models to identify how the interdependence among the variables evolve over time. Our paper builds on these methodologies, however, we extend the literature by studying higher-order moment connectedness across markets during the period from 2020 to 2022, thereby uncovering the interlinkage among the cryptocurrency, stock and commodity markets in response to recent events.

5 The connectedness results are implemented by the ConnectednessApproach R code (https://davidgabauer.shinyapps.io/connectedness_approach/).

6 The network topology is illustrated by Gephi algorithm (https://gephi.org/).

7 We focus on Bitcoin and Ethereum because these are the two largest cryptocurrencies in terms of market capitalizations. Moreover, we focus on the Eurozone, London, Japan and U.S. stock markets as these markets represent the most developed financial markets globally. Finally, our data set also includes two commodities, oil and gold, since these assets have been found to be closely linked to stock and cryptocurrency markets (e.g. Nakagawa and Sakemoto 2022; Elysayed et al. 2022; Benlagha and El Omari 2022; Nham 2022; Hung and Vo 2021; Coronado et al 2018, among many others). The high frequency of trading in these markets also ensures sufficient variations in our data for the calculation of the higher-moment measures.

8 For example, China and 8 other countries have banned cryptocurrencies. See https://fortune.com/2022/01/04/crypto-banned-china-other-countries/.

9 A window of 200 days corresponds to a trading year. This is long enough to eliminate any short-term shocks that may distort the true relationship among variables, while still being short enough to capture any changes in the spillovers across variables. Our results are robust to other window length such as 150 and 250 days.
